# Ticks of Kazakhstan: a systematic review, evidence-based taxonomic checklist, and assessment of molecular confirmation gaps

**DOI:** 10.3389/fvets.2026.1974724

**Published:** 2026-10-28

**Authors:** Kairat Zhumanov, Ainur Nurpeisova, Zhandos Abay, Daerhan Maidanniyati, Serik Khizat, Mynbay Umitzhanov, Saltanat Nussupova, Zaure Sayakova, Uinkul Izbanova, Akhmetzhan Sultanov, Nurkuisa Rametov, Wurelihazi Hazihan, Abylay Sansyzbay, Markhabat Kassenov, Fariza Ikramkulova, Raikhan Nissanova

**Affiliations:** 1 NJSC Kazakh National Agrarian Research University, Almaty, Kazakhstan; 2 College of Animal Science and Technology, Shihezi University, Shihezi, China; 3 Kazakh Scientific Research Veterinary Institute LLP, Almaty, Kazakhstan; 4 M. Aikimbayev’s National Scientific Center for Especially Dangerous Infections, Almaty, Kazakhstan; 5 Research and Development Department, Tecton Analytics LLP, Astana, Kazakhstan

**Keywords:** Argasidae, biodiversity, Central Asia, DNA barcoding, Ixodida, Kazakhstan, systematic review, taxonomy

## Abstract

**Background:**

Published estimates of Kazakhstan’s tick diversity vary because historical names, synonyms, regional records, unverified range statements, and unevenly sampled modern datasets have often been combined without consistent evidence criteria. We aimed to produce an auditable national checklist that separates verified occurrence from provisional, unresolved, and non-independent records.

**Methods:**

We systematically reviewed English-, Russian-, and Kazakh-language sources reporting ticks from the present-day territory of Kazakhstan, with no lower publication-date limit and with searches conducted through 28 July 2026. Historical monographs, field surveys, molecular studies, collection-based records, and taxonomic works were evaluated at the taxon–locality–evidence level. Original names were retained and reconciled with current taxonomy. Records were graded according to locality and specimen traceability, identification method, molecular evidence for the tick itself, and availability of sequence or voucher information.

**Results:**

The evidence database contained 62 source records and 206 taxon–locality evidence records. After reconciliation of 59 source-name entries, 42 species in ten genera and two families were retained: 23 met the strict criteria for verified occurrence and 19 were provisionally verified. Ten additional currently valid taxa were presented separately because their Kazakhstan occurrence requires primary-source confirmation, specimen re-examination, or taxonomic reassessment. Recently strengthened records included *Hyalomma dromedarii, Dermacentor nuttalli, D. niveus, Ixodes occultus, I. crenulatus, Haemaphysalis concinna, Rhipicephalus annulatus,* and *Argas reflexus*. Molecular identification of the tick was available for 13 of 23 verified species (56.5%) and 14 of all 42 retained species (33.3%). Evidence remained geographically and host-biased despite improved coverage of western and northern Kazakhstan.

**Conclusion:**

Kazakhstan’s tick fauna cannot be represented reliably by a single inherited species total. The updated evidence-graded checklist provides a reproducible baseline for taxonomy, biodiversity assessment, veterinary and public-health surveillance, and targeted voucher-based molecular resampling in Kazakhstan and across Central Asia.

## 1 Introduction

Ticks are obligate blood-feeding arthropods that link wildlife, livestock, companion animals, and humans across natural and managed landscapes (1, 2). Their importance extends beyond their role as vectors: tick distributions are shaped by host communities, climate, vegetation, land use, animal movement, and historical patterns of faunal exchange (3–5). Kazakhstan is particularly informative in this context. Its territory connects Eastern Europe, western Siberia, Central Asia, and north-western China and encompasses forest-steppe, steppe, semi-desert, desert, mountain, and river-valley ecosystems (6–9). This environmental heterogeneity allows northern, arid-zone, and montane tick assemblages to overlap, while extensive livestock production and transboundary animal movement create a practical need for accurate species-level surveillance (10, 11).

Knowledge of the tick fauna of Kazakhstan has a substantial historical foundation (12–14). The five-volume monographic series Blood-sucking Ticks of Kazakhstan by I. G. Galuzo, published between 1946 and 1953, remains the principal early synthesis of tick morphology, host associations, distribution, and ecology in the country (15). Subsequent Soviet and post-Soviet studies contributed regional occurrence records, host data, descriptions of new taxa, and epidemiological observations. Although these sources remain scientifically valuable, their records cannot be transferred directly into a contemporary checklist. Historical species concepts may differ from those currently accepted, generic combinations have changed, several names have been synonymized or reinterpreted, and some records have been repeated in later publications without re-examination of the original material.

Published estimates of tick diversity in Kazakhstan are therefore inconsistent. Values reported in monographs, reviews, regional surveys, and introductory summaries may represent fundamentally different units, including nominal taxa, currently accepted species, regional species lists, both *ixodid* and *argasid* ticks, or members of *Ixodidae* alone. Comparing these values without tracing individual names to their primary evidence risks both inflation and underestimation of national diversity. Synonyms may be counted as separate species, whereas valid but infrequently collected species may be omitted from recent summaries. Moreover, an occurrence supported by examined specimens from a defined locality is not equivalent to a generalized distribution map, a secondary checklist, or a statement that a species is expected to occur in Central Asia. Regional surveys, formal species descriptions, pathogen-oriented investigations, and broader post-Soviet syntheses illustrate the heterogeneous forms in which evidence from Kazakhstan has been reported (16–19).

Molecular approaches have improved the resolution of selected records. Studies employing mitochondrial cytochrome c oxidase subunit I (COI), 16S rRNA, internal transcribed spacer 2, and other markers have characterized several *ixodid* and *argasid* species collected in southern, eastern, and south-eastern Kazakhstan (20–23). These data can support morphology-based identification, reveal intraspecific genetic structure, and facilitate comparison of records across national borders. Molecular coverage, however, remains uneven among taxa, administrative regions, host groups, and life stages (13, 24).

A 2020 review synthesized the available information on tick-borne pathogens and their vectors in Kazakhstan and demonstrated the breadth of the relevant literature (14). Its principal objective, however, was not a detailed taxonomic reassessment of the national tick fauna. Since its publication, additional DNA-barcoding studies and occurrence records have become available, while the global classification and nomenclature of ticks have continued to evolve (25). An updated national synthesis must therefore integrate historical zoological literature, contemporary field surveys, molecular studies, collection-based evidence, and current taxonomic concepts within a transparent and reproducible framework.

The central methodological challenge is not simply to retrieve every species name associated with Kazakhstan, but to determine what each reported name actually demonstrates. For every candidate taxon, the evidence chain should establish who reported it, where and when the material was collected, what specimens or other material were examined, how identification was performed, and how the name used in the source corresponds to the currently accepted taxon (26–28). This approach allows verified occurrences to be distinguished from provisional identifications, historical records requiring contemporary confirmation, questionable reports, range-only statements, and names that should not be counted independently.

Accordingly, this study aimed to develop, to our knowledge, the first systematic and evidence-graded national checklist of ticks reported from Kazakhstan. The study was designed to critically evaluate historical and contemporary occurrence records without imposing a lower publication-date limit, distinguish primary occurrence records from secondary citations and generalized range statements, and reconcile source-reported names with current taxonomy. It also aimed to assess the geographical, host, temporal, and methodological coverage of the available evidence, establish which accepted taxa were supported by molecular identification of the tick itself, and define priorities for renewed sampling and taxonomic reassessment. The overarching goal was to provide an auditable framework for biodiversity research, collection curation, vector surveillance, and targeted voucher-based molecular investigation, rather than to reproduce another unqualified species total from previous summaries.

## 2 Methods

### 2.1 Study design and reporting framework

This study was conducted as a systematic review with an evidence-graded taxonomic checklist as its principal output. The systematic-review framework was used to ensure transparent and reproducible source identification, screening, duplicate control, and evidence selection rather than to replace conventional faunistic or taxonomic appraisal. Taxonomic reconciliation and checklist construction remained the principal zoological outputs and were based on primary occurrence evidence, historical and collection material, morphology, type-locality information, and molecular identification where available. The review was reported in accordance with the Preferred Reporting Items for Systematic Reviews and Meta-Analyses 2020 statement (PRISMA 2020) (29), while the search procedures were documented following the PRISMA extension for literature searches (PRISMA-S) (30). Relevant elements of the Joanna Briggs Institute guidance for evidence mapping and scoping reviews were also applied where they improved the transparency and reproducibility of the review process (31). A dated review protocol, eligibility criteria, and complete search strategies were finalized before formal title and abstract screening and retained as part of the project documentation. Any subsequent methodological amendments were recorded in the review log and reported when they could influence the interpretation of the findings.

### 2.2 Review questions

The review examined which tick species had been reported from the present-day territory of Kazakhstan and whether each report was supported by primary occurrence evidence. Particular attention was given to the relationship between names used in historical sources and currently accepted taxa, including changes in generic combinations, synonymy, probable misidentifications, and taxonomically unresolved records. Each candidate taxon was evaluated to determine whether the available evidence supported its classification as verified, provisionally verified, historically supported but requiring confirmation, questionable, or attributable to synonymy or identification error. The review also characterized the geographical, host, temporal, and methodological distribution of reliable records across Kazakhstan. Molecular evidence was assessed separately to determine which accepted species had been confirmed through molecular identification of the tick itself and to identify the principal taxonomic and geographical gaps requiring renewed sampling or re-examination.

### 2.3 Eligibility criteria

Eligibility was determined according to geographical relevance, evidentiary basis, taxonomic scope, identification method, publication type, and retrievability of the reported information. Records were eligible for evidence synthesis when they provided Kazakhstan-specific information traceable to collected or examined tick material. Secondary sources were used for reference chaining and contextual interpretation but were not treated as independent evidence of species occurrence (Table 1).

**Table 1:** Eligibility criteria applied in the systematic review and evidence-graded checklist.

Domain	Include	Exclude	Reason for decision
Geography	Records from the present-day territory of Kazakhstan with a stated locality, administrative area, coordinates, or collection context that can be assigned to the country.	Records only from neighboring countries; broad Central Asian or Palearctic range statements without Kazakhstan-specific material.	Prevents inferred range from being treated as observed national occurrence.
Evidence	Primary reports of collected or examined ticks; collection catalogues with specimen data; taxonomic descriptions; molecular studies; historical works reporting traceable material.	Secondary repetition without access to or citation of identifiable primary evidence; unsupported species lists.	Preserves an auditable chain from checklist entry to evidence.
Taxa	All tick families and life stages reported from Kazakhstan, including historical names and candidate synonyms.	Mites outside Ixodida; pathogen detections with no usable tick identification.	Defines the faunal scope while separating vector and pathogen identification.
Identification	Morphological, molecular, integrative, or expert collection identification, provided the method is described or inferable from the primary source.	Common names alone; genus-only records for species-level checklist decisions.	Allows method-specific evidence grading.
Publication	Peer-reviewed articles, monographs, books, dissertations, institutional series, collection catalogues, official reports, and relevant conference material with sufficient data.	News items, commercial pages, unsourced web lists, and inaccessible citations with no recoverable evidence.	Captures historical grey literature without abandoning minimum verifiability.
Language/date	English, Russian, and Kazakh; other languages when an interpretable record is retrievable. No lower date limit.	No exclusion solely by year. Language exclusion only when the record cannot be reliably interpreted.	Reduces historical and language bias.

### 2.4 Information sources and search strategy

Web of Science Core Collection, Scopus, PubMed/MEDLINE, Zoological Record, CAB Abstracts, Crossref, and Google Scholar were searched from their respective dates of coverage through 28 July 2026. Russian-language and regional literature was identified through eLIBRARY. RU, CyberLeninka, Kazakhstan-based institutional repositories and library catalogues, the Zoological Institute of the Russian Academy of Sciences, and the Institute of Zoology of the Republic of Kazakhstan. All five volumes of Galuzo’s Blood-sucking Ticks of Kazakhstan were examined directly (32). Backward and forward citation searching was performed for included sources and relevant reviews, and collection catalogues were examined where available.

Search strategies combined English, Russian, and Kazakh geographical terms with tick-related terms at the order, family, genus, species, and common-name levels. Historical spellings, transliterations, synonyms, and legacy generic combinations were incorporated during iterative taxon-specific searches. Pathogen terms were not mandatory because many relevant faunistic and taxonomic publications did not report pathogens. Google Scholar results were screened in relevance-ranked blocks of 100 until two consecutive blocks yielded no additional eligible sources. Complete platform-specific search strings, search dates, applied limits, and numbers of retrieved records are provided in Supplementary Table S1.

### 2.5 Record management and study selection

Search results were exported with complete bibliographic metadata and deduplicated using DOI, PMID, title, year, author, file checksum, and manual comparison of transliterated records. The review unit was a source, whereas the checklist unit was a taxon–locality–evidence record. A source record represented one retrievable publication or other source; an evidence record represented one taxon–locality–evidence unit; a source-name entry represented a reported taxon name carried into taxonomic reconciliation; and an accepted species represented the current taxon resulting from that reconciliation. Multiple records extracted from one publication remained linked to that source; exact duplicate files and derivative reports were not counted as independent evidence. Two reviewers independently screened titles and abstracts and then assessed the full texts. Records retained because the title or abstract was ambiguous proceeded to full-text assessment. Disagreements were resolved by discussion and, when needed, a third reviewer. Reasons for exclusion at full-text assessment were recorded using a controlled vocabulary; aggregate exclusion categories and counts, together with representative report-level audit entries, are provided in Supplementary Table S1. Russian and Kazakh sources were screened in the original language; machine-assisted translation supported searching but did not replace verification of the taxonomic statement in the source.

### 2.6 Data extraction and verification

Data were extracted using a standardized form recording bibliographic details, the reported and accepted taxon names, locality, collection period, specimen number, host or substrate, identification method, molecular marker and accession number, evidence level, and checklist decision. One reviewer performed the extraction, and a second reviewer verified the records against the original sources; disagreements were resolved by consensus. Coordinates reported in the source were distinguished from those georeferenced from named localities. Georeferenced records retained their source and estimated uncertainty, whereas records available only at the administrative-region level were assigned to polygons rather than artificial centroid points. Numerical inconsistencies were checked against the original text and tables and were retained with an explanatory note when they could not be resolved.

### 2.7 Taxonomic reconciliation

Taxon names were retained as reported in the original sources, while accepted names were assigned according to the current global classification of ticks and relevant primary revisions (25). Synonyms, recombined names, and spelling or transliteration variants were not counted as separate species. Historical names were linked to accepted taxa only when supported by an authoritative taxonomic source and compatible with the original record; ambiguous names, unresolved species complexes, and open-nomenclature records were retained as unresolved. All decisions were documented in a reconciliation table containing the source name, accepted name, taxonomic action, authority, and rationale. Type-locality and type-specimen information was recorded for taxa described from Kazakhstan.

### 2.8 Evidence appraisal and checklist decisions

Evidence strength was assessed at the record level using the framework shown in Table 2. The A–E levels were not intended as a linear ranking of identification methods; in particular, molecular identification was not automatically weighted above well-documented morphological, integrative, or type-material evidence. Molecular confirmation was assigned only when tick-derived DNA was analyzed and the marker, sequence accession, or diagnostic method was reported. Molecular detection of a pathogen in a morphologically identified tick did not constitute molecular confirmation of the tick species. Evidence level and checklist status were treated as related but distinct variables. Evidence levels A–E describe the strength of individual occurrence records, whereas the final checklist decision was assigned at the accepted-species level using the strongest qualifying record together with the consistency of the remaining evidence. For records in which more than one checklist outcome was possible, the decision was based on four modifiers: traceability of the Kazakhstan locality and examined material; adequacy of the reported identification basis; stability of the taxonomic assignment under current classification; and independent corroboration by additional occurrence records or type/voucher material. Molecular sequence quality and accession information were additionally considered when available. Specimen number, life stage, and other diagnostic details were treated as supporting information when reported; missing information was not inferred and could not increase the checklist status.

**Table 2:** Evidence framework and corresponding checklist decisions.

Level	Minimum evidence	Typical interpretation	Default contribution to species-level checklist decision
A	Kazakhstan locality plus molecular identification of the tick; preferably sequence accession and voucher or retained material.	Strong, independently traceable modern evidence.	Verified, unless sequence quality or unresolved taxonomy prevents an unambiguous species assignment.
B	Exact or assignable Kazakhstan locality plus reliable modern morphological or integrative identification and described material.	Strong occurrence evidence without tick sequence confirmation.	Verified when the identification is adequately documented and the occurrence is independently corroborated or supported by type/voucher material; otherwise Provisionally verified.
C	Historical primary record with locality and examined or collected material, but limited modern diagnostic detail.	Direct evidence that requires historical taxonomic interpretation.	Provisionally verified when the primary historical record is directly traceable and its name can be reconciled unambiguously with a currently accepted species; Pending taxonomic resolution when such reconciliation remains uncertain.
D	Secondary statement, imprecise locality, untraceable identification, or ambiguous name/material.	Insufficient for an unqualified national occurrence claim.	Pending primary-source confirmation when a plausible occurrence lacks recoverable primary evidence; otherwise Excluded until verified.
E	Synonym counted separately, demonstrated error, unsupported range-only statement, or record outside Kazakhstan.	No independent national species evidence.	Not counted separately when representing synonymy, obsolete usage, demonstrated error, or a non-independent occurrence claim.

Where several records were available for the same accepted species, a weaker record did not override stronger evidence, but conflicting taxonomic or identification evidence could prevent assignment to Verified status. Accordingly, checklist status reflected both the strongest qualifying evidence and the absence of unresolved contradictory evidence. The final evidence synthesis used six mutually exclusive outcomes: Verified, Provisionally verified, Pending primary-source confirmation, Pending taxonomic resolution, Excluded until verified, and Not counted separately. Only Verified species were included in the strict contemporary count; Verified and Provisionally verified species together formed the broader retained checklist.

### 2.9 Data synthesis and visualization

The synthesis was descriptive. Included sources and evidence records were summarized by publication period, source type, taxonomic group, checklist category, administrative region, host group, and identification method. Observed species richness was calculated by administrative region using unique accepted taxa. Geographic records were mapped at the most precise defensible resolution: verified coordinates were represented as points, whereas region-level records were assigned to administrative polygons. Georeferenced coordinates were distinguished from coordinates reported in the original source. For descriptive purposes, molecular coverage was calculated as a binary availability measure: an accepted species was counted as having molecular identification when at least one qualifying tick-derived molecular record was available. This measure was not intended to imply equivalent strength or quality of molecular evidence among species. Sensitivity analyses compared the strict count of Verified species with counts additionally including Provisionally verified or historical records. No prevalence meta-analysis was undertaken because sampling frames, host populations, collection methods, seasons, and denominators were highly heterogeneous. Counts from different publications were not pooled unless the underlying specimens were demonstrably independent.

### 2.10 Missing information and protocol deviations

Missing information was coded as not reported and was not inferred. Unresolved ambiguity resulted in a lower evidence classification rather than an unsupported assignment. Methodological deviations from the predefined protocol were documented and justified. The source list, evidence dataset, taxonomic reconciliation table, and final checklist are provided as Supplementary materials.

## 3 Results

### 3.1 Evidence corpus and checklist disposition

The evidence database contained 62 source records, from which 206 taxon–locality evidence records and 59 source-name checklist entries were extracted. The evidence records comprised 30 records with molecular confirmation of tick identity, five with supplementary molecular information that did not independently establish species-level tick identity, 153 morphology-based or supporting-morphology records, two type-locality records, and 16 records representing new or possible occurrences, historical evidence, synonymy, taxonomic uncertainty, or rejected records. These categories describe the type of evidence contained in individual records and are distinct from the A–E evidence framework. Potentially overlapping field series remained source-linked and their specimen counts were not pooled. Following taxonomic reconciliation, 23 species belonging to seven genera and two families met the strict inclusion criteria. A further 19 species were retained provisionally, resulting in a broader evidence-supported set of 42 species in ten genera and two families. Thirteen of the 23 verified species had molecular confirmation of tick identity (56.5%); when provisional species were included, molecular coverage was 14 of 42 species (33.3%). To improve taxonomic transparency, Table 3 additionally presents ten currently valid taxa requiring primary-source confirmation, specimen re-examination, or taxonomic reassessment; these taxa are not included in the retained 42-species total. The study-selection process leading to the final corpus of 62 source records is summarized in Figure 1.

**Table 3:** Evidence-based checklist and taxonomic status of currently valid tick taxa reported from Kazakhstan.

Family	Accepted species	Checklist category	Molecular confirmation	Principal source(s)
Ixodidae	*Hyalomma asiaticum*	Verified	Yes	(20, 22, 23, 28, 34)
Ixodidae	*Hyalomma anatolicum*	Verified	Yes	(42, 49)
Ixodidae	*Hyalomma scupense*	Verified	Yes	(13, 20, 23, 38, 49)
Ixodidae	*Hyalomma marginatum*	Verified	Yes	(21, 23, 50)
Ixodidae	*Hyalomma dromedarii*	Verified	No	(37)
Ixodidae	*Hyalomma excavatum*	Provisionally verified	No	(37, 38)
Ixodidae	*Hyalomma rufipes*	Provisionally verified	No	(51)
Ixodidae	*Hyalomma turanicum*	Provisionally verified	No	(49)
Ixodidae	*Dermacentor marginatus*	Verified	Yes	(19, 42)
Ixodidae	*Dermacentor reticulatus*	Verified	Yes	(19, 42)
Ixodidae	*Dermacentor pavlovskyi*	Verified	No; type-locality evidence	(15)
Ixodidae	*Dermacentor ushakovae*	Verified	No; type-locality evidence	(52)
Ixodidae	*Dermacentor nuttalli*	Verified	No	(35)
Ixodidae	*Dermacentor niveus*	Verified	Yes	(19, 23, 37)
Ixodidae	*Ixodes persulcatus*	Verified	Yes	(17, 18, 38)
Ixodidae	*Ixodes kazakstani*	Verified	No Kazakhstan sequence	(16)
Ixodidae	*Ixodes occultus*	Verified	No	(36)
Ixodidae	*Ixodes crenulatus*	Verified	No	(36)
Ixodidae	*Ixodes ricinus*	Provisionally verified	Not established	(21, 36, 51)
Ixodidae	*Ixodes laguri*	Provisionally verified	No	(37, 41)
Ixodidae	*Ixodes pavlovskyi*	Provisionally verified	No Kazakhstan sequence	(16)
Ixodidae	*Ixodes stromi*	Provisionally verified	No	(53)
Ixodidae	*Haemaphysalis punctata*	Verified	Yes	(17, 35, 38)
Ixodidae	*Haemaphysalis erinacei*	Verified	Yes	(35, 42)
Ixodidae	*Haemaphysalis concinna*	Verified	No	(35)
Ixodidae	*Haemaphysalis sulcata*	Provisionally verified	Yes	(18, 23, 33, 49)
Ixodidae	*Haemaphysalis pospelovashtromae*	Provisionally verified	No	(54)
Ixodidae	*Rhipicephalus turanicus*	Verified	Yes	(17, 22, 49)
Ixodidae	*Rhipicephalus annulatus*	Verified	Yes	(23, 49, 51)
Ixodidae	*Rhipicephalus schulzei*	Provisionally verified	No	(37, 41, 55)
Ixodidae	*Rhipicephalus rossicus*	Provisionally verified	No	(41, 51, 55)
Ixodidae	*Rhipicephalus bursa*	Provisionally verified	No	(41)
Ixodidae	*Rhipicephalus pumilio*	Provisionally verified	No	(37, 41, 51)
Argasidae	*Argas persicus*	Verified	Yes	(18, 38)
Argasidae	*Argas reflexus*	Verified	No	(18)
Argasidae	*Argas vulgaris*	Provisionally verified	No	(39)
Argasidae	*Ornithodoros tartakovskyi*	Verified	No	(18, 37, 41)
Argasidae	*Alveonasus lahorensis*	Provisionally verified	No	(15, 25)
Argasidae	*Carios vespertilionis*	Provisionally verified	No	(39)
Argasidae	*Ornithodoros papillipes*	Provisionally verified	No	(56, 57)
Argasidae	*Alectorobius coniceps*	Provisionally verified	No	(58)
Argasidae	*Argas tridentatus*	Provisionally verified	No	(39)
Currently valid taxa requiring validation or taxonomic reassessment (not included in the retained 42-species total)				
Ixodidae	*Rhipicephalus leporis*	Need validation	No	(25, 41)
Argasidae	*Alectorobius capensis*	Need validation	No	(25, 39)
Ixodidae	*Dermacentor silvarum*	Need validation	No	(19)
Ixodidae	*Dermacentor raskemensis*	Need validation	No	(17, 26)
Ixodidae	*Dermacentor kamshadalus*	Need validation	No	(17, 26)
Ixodidae	*Ixodes redikorzevi*	Need validation	No	(41)
Ixodidae	*Ixodes lividus*	Need validation	No	(12)
Ixodidae	*Haemaphysalis parva*	Need taxonomic reassessment	No	(26)
Ixodidae	*Rhipicephalus sanguineus s.s.*	Need taxonomic reassessment	No	(12, 15, 38)
Argasidae	*Ornithodoros cholodkovskyi*	Need validation	No	(39)

Species are grouped according to the strength and interpretation of the available Kazakhstan evidence. Verified and provisionally verified species constitute the retained evidence-supported checklist, whereas currently valid names requiring primary-source confirmation, specimen re-examination, or taxonomic resolution are shown separately for transparency and are not included in the retained 42-species total. Reference numbers in the final column identify the principal occurrence or taxonomic source(s). Species-level evidence and detailed decision rationale are provided in Supplementary Table S2.

**Figure 1:**
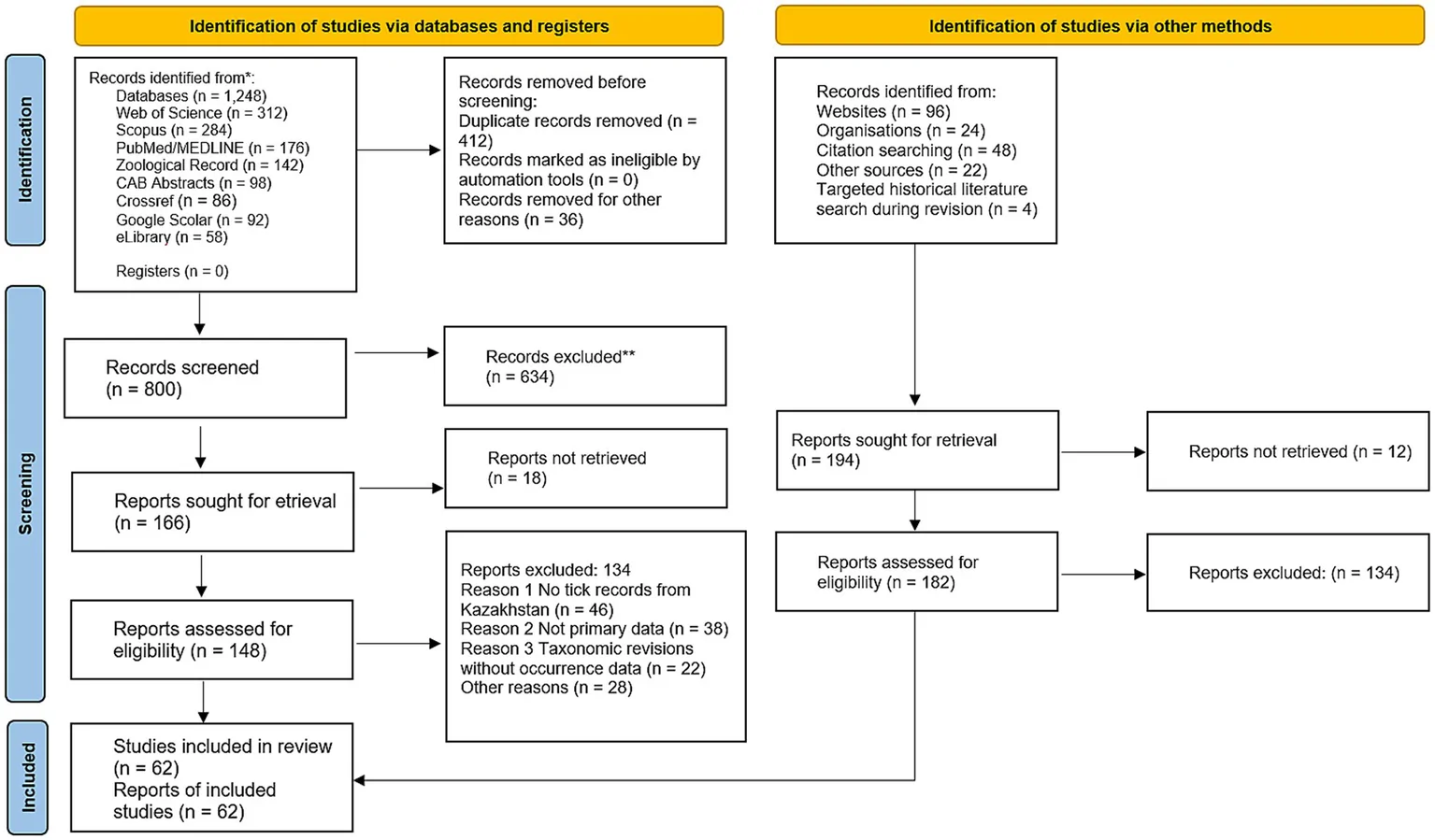
PRISMA 2020 flow diagram of study selection for the systematic review. The diagram summarizes record identification through databases and other sources, duplicate removal, screening, report retrieval, full-text eligibility assessment, reasons for exclusion, and the inclusion of 62 source records in the final evidence synthesis. *Consider, if feasible to do so, reporting the number of records identified from each database or register searched (rather than the total number across all databases/registers). **If automation tools were used, indicate how many records were excluded by a human and how many were excluded by automation tools. Source: Page et al. (59). This work is licensed under CC BY 4.0. To view a copy of license, visit https://creativecommons.org/licenses/by/4.0/

### 3.2 Characteristics of the evidence base

Dated source records spanned 1946–2026, with 43 of 61 dated records (70.5%) published between 2020 and 2026; one source was undated. Of the 206 taxon–locality evidence records, 30 (14.6%) provided molecular confirmation of tick identity, five (2.4%) provided additional molecular support, 153 (74.3%) were morphology-based, two (1.0%) were supported by type-locality material, and 16 (7.8%) represented new or possible occurrences, historical, synonym-related, taxonomically uncertain, or rejected evidence. Geographic coverage remained concentrated in southern, south-eastern, and eastern Kazakhstan, but recent datasets added substantial evidence from western Kazakhstan, the Irtysh floodplain, northern Aktobe Region, and East Kazakhstan (Figure 2).

**Figure 2:**
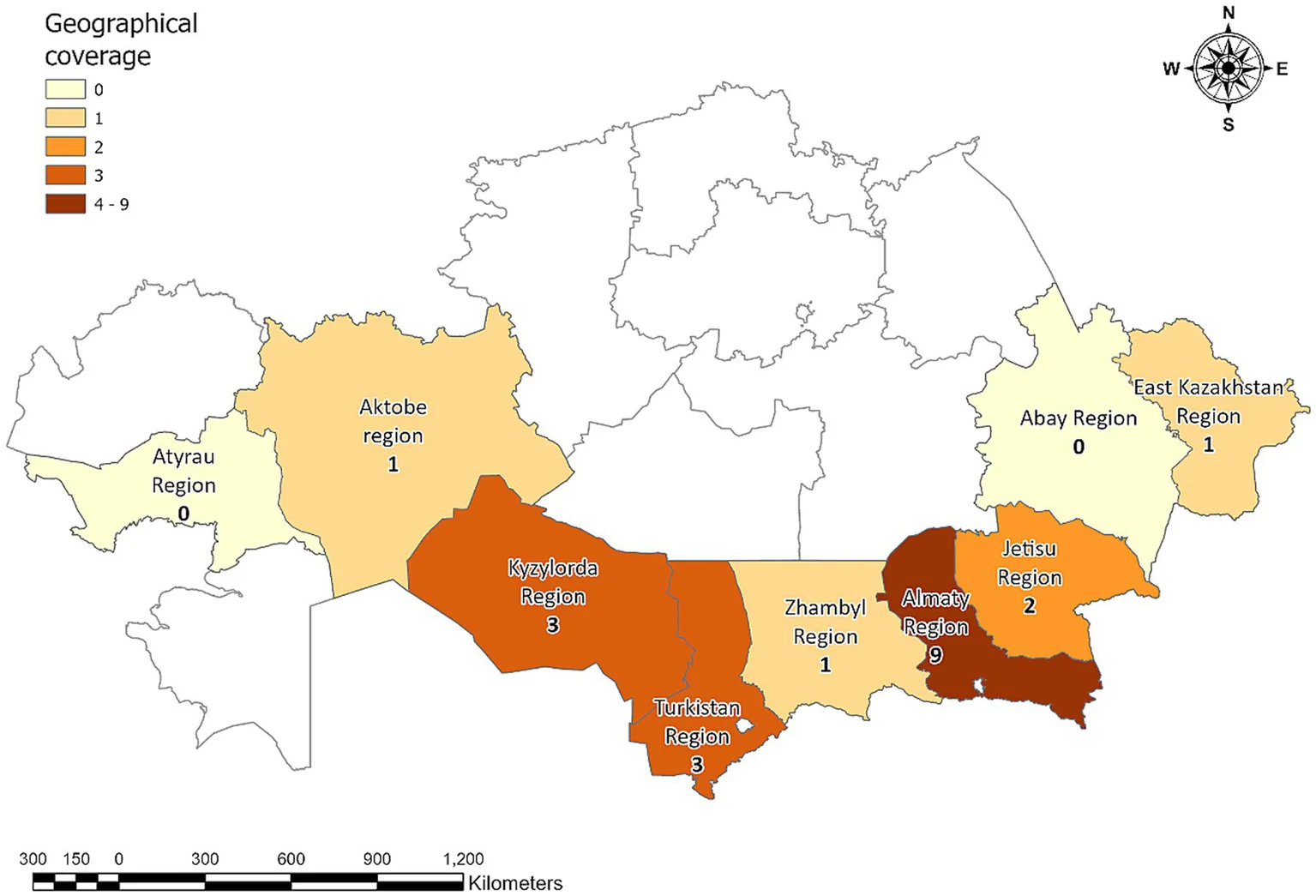
Geographic coverage of the evidence base included in the systematic review. Administrative regions are shaded according to the availability of qualifying occurrence evidence retrieved during the systematic review. The map illustrates the spatial distribution of the evidence base rather than the true distribution or diversity of ticks. Areas with greater evidence density reflect more intensive sampling and publication effort, whereas regions with limited or no records indicate current evidence gaps and priorities for future surveys. This figure provides the geographical context for the evidence synthesis and should not be interpreted as a measure of species richness or sampling completeness.

Dedicated sampling in central Kazakhstan and in several northern, desert, mountain, and border areas remained sparse. Examined material included ticks collected from livestock, wild mammals, poultry, vegetation, burrows, shelters, and other habitats. Morphology was the predominant identification method; COI/cox1, 16S, or ITS2 was applied only to selected taxa or specimen subsets.

#### 3.2.1 Modern field datasets and numerical validation

The 2021–2026 update added several large morphology-based datasets, including 100,539 Hyalomma ticks from southern Kazakhstan, 15,042 ticks from northern Aktobe Region, 14,218 from Kyzylorda Region, 4,501 from six western and southern regions, 5,928 from Turkistan Region, and 683 georeferenced ticks from the Irtysh floodplain (33–37). These source-level denominators were not pooled because sampling periods, regions, hosts, and collection designs differed and some author series probably overlapped. An additional integrative study provided morphology- and COI-based confirmation of *H. scupense* and *H. asiaticum* from Kyzylorda, Zhambyl, Turkistan, and Jetisu regions (20). A further 2025 study analyzed 3,121 ticks collected from cattle in Almaty, Zhambyl, and Turkistan regions and provided cox1-based confirmation of *H. scupense*, *H. marginatum*, *H. asiaticum*, *D. niveus*, *Hae. sulcata*, and *R. annulatus* (23).

For the 19,861-tick dataset, taxon-specific counts summed to the stated denominator, but published percentages for *H. scupense*, *Dermacentor marginatus*, and *H. asiaticum* differed from values recalculated from the reported counts: 45.49% versus 44.09%, 20.38% versus 20.07%, and 12.49% versus 13.90%, respectively (38). The Irtysh floodplain study also contained an unresolved discrepancy in the reported number of *D. marginatus* (35). These differences were retained as source-level data-quality findings rather than silently corrected.

#### 3.2.2 Historical evidence and taxonomic decisions from Galuzo Volume III

A full-text audit was conducted for Volume III of Galuzo’s monographic series, which covers *Dermacentor* and *Rhipicephalus* (32). Because the volume incorporates material from Kazakhstan and other parts of the former USSR, it was not interpreted as a Kazakhstan-specific checklist. The 16 names included in its systematic index were treated as search leads; only species accounts containing a Kazakhstan locality or traceable specimen information were accepted as occurrence evidence.

Three records materially affected checklist decisions. *Dermacentor pavlovskyi* was supported by a type series of two males and one female collected on 31 January 1910 in the Karatau Range, Ak-Kenil tract, then within the Syr-Darya Region, with museum number 43–1910 reported by Galuzo (15). This type-locality evidence supported verification of the species in Kazakhstan, although modern population-level molecular confirmation remains unavailable. *Dermacentor daghestanicus* was retained as a historical source name but was not counted independently because subsequent revisions placed it inconsistently within the *D. niveus–D. marginatus* complex (18, 25, 32). Earlier Kazakhstan records of *Rhipicephalus sanguineus* were explicitly rejected by Galuzo after the species could not be recovered during repeated collections and were therefore classified as rejected historical evidence. A subsequent morphology-based record reported as *R. sanguineus sensu lato* remains taxonomically unresolved and was not assigned to *R. sanguineus* sensu stricto in the absence of barcode or lineage-level evidence (38).

### 3.3 Evidence-based national checklist

Taxonomic reconciliation of 59 source-name entries resulted in 42 species from ten genera and two families being retained in the evidence-based national checklist (Table 3). Twenty-three species met the strict criteria for verified occurrence, whereas 19 were classified as provisionally verified. Recently strengthened records included *H. dromedarii*, *D. nuttalli*, *D. niveus*, *I. occultus*, *I. crenulatus*, *Hae. concinna*, *R. annulatus*, and *A. reflexus* (18, 23, 35, 37). Ten additional currently valid taxa requiring primary-source confirmation, specimen re-examination, or taxonomic reassessment are presented separately in Table 3 and are not included in the retained 42-species total. Verified and provisionally verified species are reported separately in Table 3.

Principal sources represent primary occurrence records, type material, or directly traceable collection evidence; secondary sources are used only for taxonomic context and are not treated as independent occurrence evidence.

### 3.4 Synonymy, reclassification, and unresolved records

Six source-name entries were not counted independently because they represented established synonyms, obsolete regional usage, legacy combinations, or disputed historical concepts. *Hyalomma detritum* was reconciled to *H. scupense* and *Dermacentor pictus* to *D. reticulatus*; historical *H. plumbeum* was attributed to *H. marginatum*, and *Haemaphysalis erinacei taurica* was collapsed into *Hae. erinacei*. *Haemaphysalis otophila* was retained as a historical source name associated with *Hae. parva*; because the Kazakhstan material requires re-examination under the current species concept, *Hae. parva* is shown separately in Table 3 as requiring taxonomic reassessment rather than being included in the retained checklist. *Dermacentor daghestanicus* remained associated with the disputed *D. niveus–D. marginatus* complex and was not counted independently. The *Haemaphysalis numidiana–Hae. n. turanica* complex remained unresolved and was not automatically merged with *Hae. erinacei* in the absence of reassessment of type material and diagnostic characters.

*Rhipicephalus leporis* is currently recognized as a valid species distinct from *R. linnaei* (25). However, the Kazakhstan material reported as *R. leporis* has not been re-examined under the current species concept; its national occurrence was therefore classified as Need validation and was not included in the retained 42-species checklist. Available *R. sanguineus sensu lato* records lack lineage-level resolution and cannot be assigned confidently to *R. sanguineus* sensu stricto, which is therefore shown separately as requiring taxonomic reassessment. *Dermacentor silvarum, Ixodes lividus, I. redikorzevi,* and *Ornithodoros cholodkovskyi* are currently valid taxa for which qualifying Kazakhstan primary occurrence evidence remains insufficient or unrecovered and are displayed separately as requiring validation. *Dermacentor raskemensis* and *D. kamshadalus* are likewise valid names, but the reviewed evidence did not establish sufficiently traceable Kazakhstan occurrence records. The historical *Boophilus calcaratus* record could not be assigned confidently to the *Rhipicephalus microplus* complex and remains excluded until verified.

The argasid records required additional clarification. Filippova’s taxonomic re-examination of historical Kazakhstan material supports provisional retention of *Argas vulgaris* (39), whereas *A. reflexus* remains supported by an independent modern specimen-based record from Almaty (18). These records were therefore treated as distinct evidence lines rather than mutually exclusive taxonomic interpretations. *Alectorobius capensis* (= *Ornithodoros capensis*) is currently recognized as a valid species and has been historically reported from Barsa-Kelmes Island; because the original Kazakhstan material has not been re-examined under the current species concept, the record is shown in Table 3 as Need validation and is not included in the retained 42-species checklist (25, 39). *R. pumilio* remained provisionally retained because multiple Kazakhstan occurrence records support its presence despite the lack of modern integrative confirmation. *Hae. sulcata* also remained provisional despite Kazakhstan cox1 evidence because sequence representation is limited and uncertainty concerning species-complex boundaries persists.

### 3.5 Geographic and host coverage

Geographically resolved evidence remained concentrated in southern, south-eastern, and eastern Kazakhstan, but the updated corpus strengthened coverage in western Kazakhstan, northern Aktobe Region, the Irtysh floodplain, and the Zaisan area (34, 35, 37). Central Kazakhstan and several northern, mountain, desert, wildlife, and border systems remained underrepresented. Geographic differences in record density should therefore be interpreted primarily as differences in evidence coverage, not direct estimates of regional species richness.

Host-associated collections predominated, especially cattle, horses, sheep, and other domestic animals. Recent sources broadened coverage to *saiga*, rodents, hedgehogs, pigeons, small carnivores, vegetation, and burrows, improving evidence for *nidicolous* and wildlife-associated taxa. Nevertheless, standardized host-seeking sampling and repeated wildlife surveys remained uncommon, limiting host-independent comparisons of tick distributions (Figure 3).

**Figure 3:**
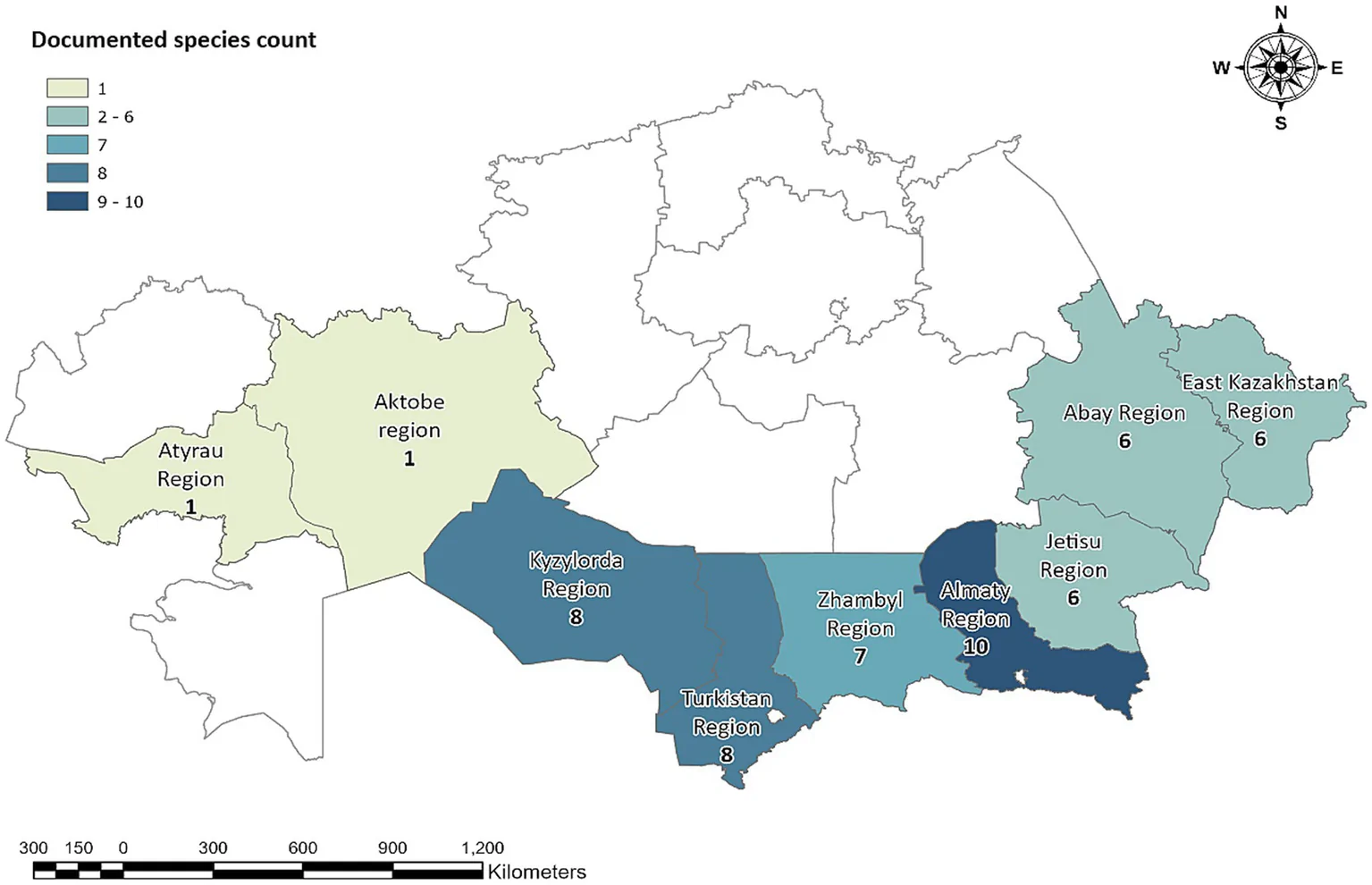
Documented species counts in the reviewed evidence across the administrative regions of Kazakhstan. Administrative regions are shaded according to the number of documented tick species supported by qualifying evidence included in this systematic review. The map reflects the current evidence base rather than standardized sampling effort; therefore, regional differences should be interpreted as differences in available evidence and not necessarily as true differences in tick species richness.

### 3.6 Molecular confirmation gaps

Molecular identification of the tick itself was available for 13 of the 23 verified species (56.5%). When the 19 provisionally verified species were included, coverage increased to 14 of 42 retained species (33.3%). These proportions describe the availability of molecular identification only; the depth and quality of the underlying molecular evidence varied among species. COI/*cox1* supported all 14 molecularly confirmed species. Published 16S- and ITS2-based data for *Ixodes kazakstani* derive from Kyrgyzstan and were therefore not counted as molecular confirmation of a Kazakhstan occurrence. The study by Sultankulova et al. (20) strengthened existing COI evidence for *H. scupense* and *H. asiaticum*, whereas Sayakova et al. (23) expanded species-level molecular coverage by confirming *H. marginatum*, *D. niveus*, *Hae. sulcata*, and *R. annulatus*.

Molecular coverage differed substantially among genera: four of eight retained *Hyalomma* species, three of six *Dermacentor*, one of eight *Ixodes*, three of five *Haemaphysalis*, two of six *Rhipicephalus*, and one of four *Argas* species had qualifying molecular evidence. No molecular confirmation was available for retained *Ornithodoros*, *Alveonasus*, *Carios*, or *Alectorobius*. Priorities for voucher-linked multilocus resampling include *H. dromedarii*, *D. nuttalli*, *I. occultus*, *I. crenulatus*, *Hae. concinna*, *A. reflexus*, and taxonomically difficult records such as *H. turanicum*, the *D. niveus–D. marginatus* complex, and members of the *R. sanguineus* group. Although *H. marginatum*, *D. niveus*, *Hae. sulcata*, and *R. annulatus* now have Kazakhstan *cox1* sequences, their limited sequence representation warrants broader voucher-linked sampling (Figure 4).

**Figure 4:**
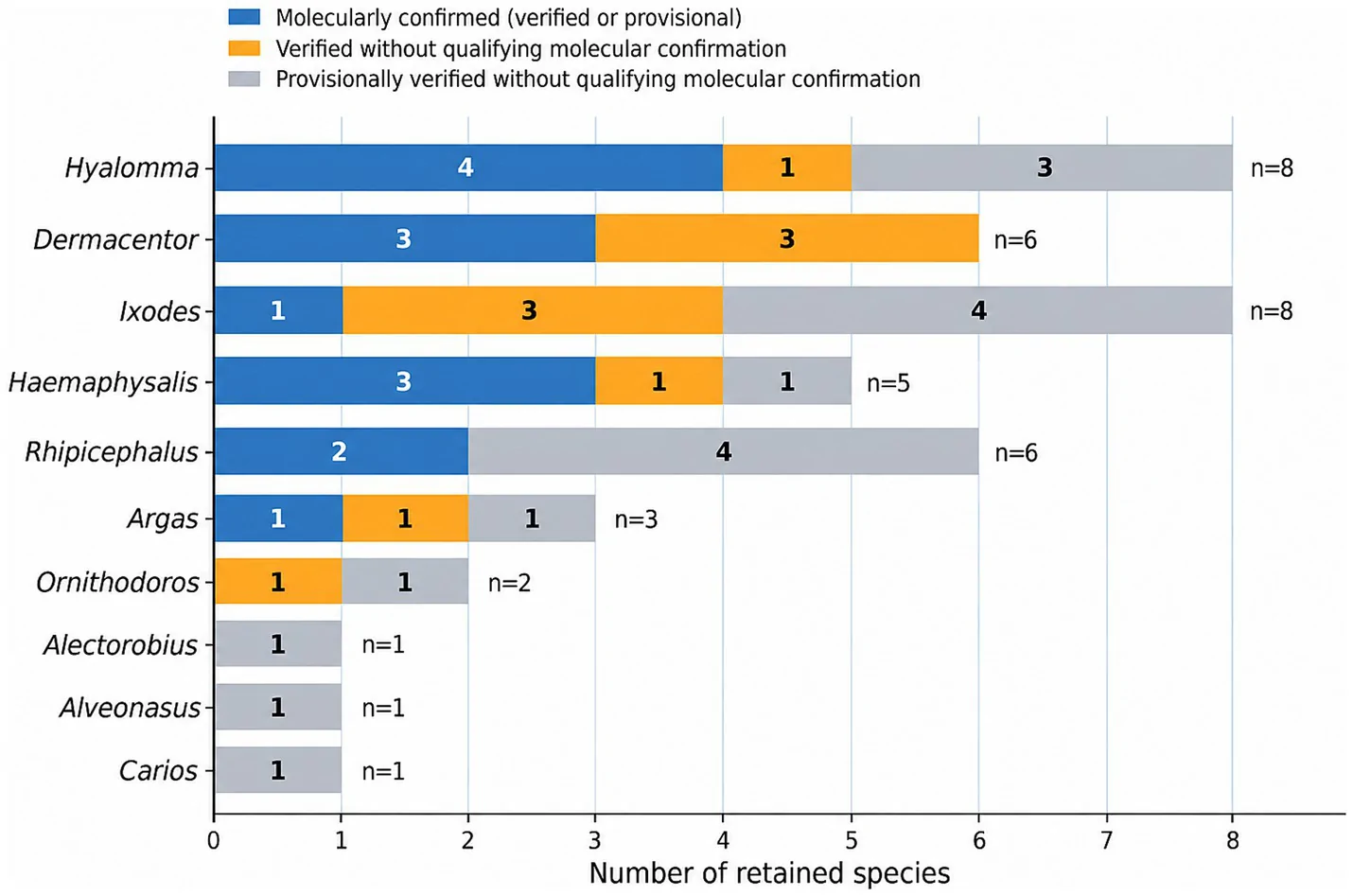
Molecular confirmation coverage and evidence gaps among tick genera retained in the national checklist of Kazakhstan. The 42 retained species comprise 14 molecularly confirmed species, 10 verified species without qualifying molecular confirmation, and 18 provisionally verified species without qualifying molecular confirmation.

## 4 Discussion

### 4.1 Principal findings

This review reconciled 59 source-name entries into an evidence-graded national checklist rather than adopting an inherited estimate of tick diversity. Under the strict rule, 23 species in seven genera and two families met the criteria for verified occurrence in Kazakhstan. Nineteen additional species were retained provisionally, producing a broader evidence-supported set of 42 species in ten genera and two families. Ten additional currently valid taxa were presented separately because their Kazakhstan occurrence requires primary-source confirmation, specimen re-examination, or taxonomic reassessment. These categories represent different levels of evidentiary certainty and should not be combined into a single unqualified species total.

The 2021–2026 update materially strengthened primary evidence for *H. dromedarii*, *D. nuttalli*, *D. niveus*, *I. occultus*, *I. crenulatus*, *Hae. concinna*, *R. annulatus*, and *A. reflexus*, while retaining *Hae. sulcata*, *H. turanicum*, *H. rufipes*, *Hae. pospelovashtromae*, and *I. stromi* provisionally because of taxonomic difficulty, rarity, or limited independent replication. Historical evidence also supported provisional inclusion of *O. papillipes*, *Alectorobius coniceps*, *A. tridentatus*, and *A. vulgaris*. Molecular identification was available for 13 of 23 verified species (56.5%) and 14 of all 42 retained species (33.3%).

### 4.2 Historical evidence

Galuzo’s monographs and subsequent Soviet zoological literature remain indispensable because they preserve historical specimens, hosts, localities, phenology, and taxonomic observations (32). Their evidentiary value is greatest when the original account provides a traceable specimen or explicit Kazakhstan locality. *Dermacentor pavlovskyi* illustrates this value: its type series and Kazakhstan type locality support verified occurrence despite the absence of modern population-level molecular data (32).

Historical names cannot be interpreted without their original context. Galuzo’s volumes included material from Kazakhstan and other parts of the former USSR, so appearance in an index or key does not establish national occurrence. Historical species concepts may also conflict with current boundaries. *Dermacentor daghestanicus* has been placed inconsistently within the *D. niveus–D. marginatus* complex (25, 32, 40), while Galuzo rejected earlier Kazakhstan records of *R. sanguineus* after repeated collections failed to recover the species. A historical record requiring confirmation therefore denotes traceable evidence that requires specimen re-examination or topotypic sampling, rather than evidence rejected because of its age.

### 4.3 Why published totals differ

Previous national estimates have varied substantially in scope, nomenclature, and evidentiary threshold. A 2020 review reported more than 30 ixodid tick species in Kazakhstan, whereas later sources cited approximately 42 and up to 50 ixodid species (14, 19, 41). These totals are not directly comparable because they were compiled under different taxonomic concepts, geographic and host coverage, and evidentiary criteria. Published totals may cover all ticks or only *Ixodidae*, the entire country or individual regions, and accepted species or historical nominal taxa. Inflation can result from counting synonyms, subspecies, alternative generic combinations, or expected geographic ranges independently. Conversely, modern surveys may omit valid but rarely collected historical taxa because sampling was restricted by host, season, habitat, or region.

The difference between the strict total of 23 verified species and the broader total of 42 verified plus provisionally verified species quantifies the effect of the evidentiary threshold. The strict estimate requires traceable modern morphological, molecular, collection, or type-locality evidence; the broader estimate also retains rare or taxonomically difficult morphology-based records that require additional vouchers, repeated sampling, or molecular confirmation. Reporting both denominators with their definitions is more informative than selecting one previous total as authoritative.

### 4.4 Molecular evidence and integrative taxonomy

Recent studies have established an important molecular reference layer, particularly through COI/*cox1* sequencing (20–23, 38, 42). COI/*cox1* supported all 14 molecularly confirmed species. Published 16S/ITS2 data for *Ixodes kazakstani* were generated from Kyrgyzstan material and were not counted as molecular confirmation of a Kazakhstan occurrence. Sultankulova et al. (20) strengthened existing evidence for *H. scupense* and *H. asiaticum*, whereas Sayakova et al. (23) provided Kazakhstan *cox1* confirmation for *H. marginatum*, *D. niveus*, *Hae. sulcata*, and *R. annulatus*.

Sequence availability does not guarantee taxonomic resolution. Its value depends on the initial morphological identification, marker choice, sequence quality, reference-database accuracy, locality metadata, and voucher availability. Conversely, a traceable specimen identified using appropriate morphology or supported by type material can provide strong occurrence evidence without DNA data. Molecular and morphological evidence should therefore be integrated rather than ranked as mutually exclusive alternatives.

Priority targets now include the strictly verified but unsequenced *H. dromedarii*, *D. nuttalli*, *D. pavlovskyi*, *D. ushakovae*, *I. occultus*, *I. crenulatus*, *Hae. concinna*, *A. reflexus*, and *O. tartakovskyi*. Although *H. marginatum*, *D. niveus*, *Hae. sulcata*, and *R. annulatus* now have Kazakhstan *cox1* sequences, the small number of sequenced specimens requires broader voucher-linked multilocus investigation (23). The *D. niveus - D. marginatus* complex and the *R. sanguineus* group require particular taxonomic caution. Future studies should link morphology, informative loci, precise locality and host metadata, and permanent vouchers to the same specimen series.

### 4.5 Geographic and surveillance implications

The spatial pattern reflected both tick ecology and research effort. The 2021–2026 update strengthened western and northern occurrence evidence and documented range extensions such *as H. asiaticum* in the Zaisan area (34) and *R. annulatus* in Almaty and Zhambyl regions (23), but southern and south-eastern livestock systems remained disproportionately represented. Record density should therefore not be interpreted directly as regional species richness.

Sampling was dominated by ticks collected from livestock, particularly cattle, horses, and sheep. Wildlife, host-seeking ticks, vegetation, shelters, and burrows were represented less consistently, potentially underestimating *nidicolous* taxa, wildlife-associated stages, and species occurring away from livestock systems. The absence of records from some regions therefore indicates insufficient documentation rather than confirmed absence. Resampling should prioritize northern and central Kazakhstan, mountain and desert ecosystems, border areas, wildlife hosts, and localities associated with unresolved historical records. Maps should distinguish source-reported coordinates, georeferenced historical localities, and administrative-region records.

### 4.6 Strengths and limitations

Strengths include the multilingual search, direct evaluation of historical and recent primary sources, preservation of original names, file-level duplicate control, and record-level separation of occurrence evidence from taxonomic interpretation. The review also retained numerical inconsistencies and potential dataset overlap as explicit quality-control annotations. By integrating historical taxonomy, biodiversity evidence, veterinary surveillance, wildlife host records, and molecular confirmation within a unified evidence framework, this review also provides a practical baseline for One Health surveillance and cross-sector vector-borne disease preparedness in Central Asia.

Completeness was limited by incomplete digitization, inaccessible dissertations or collection labels, inconsistent transliteration, changing administrative geography, and loss or inaccessibility of historical vouchers (43, 44). Modern studies remained geographically and ecologically uneven and were often designed for pathogen surveillance rather than faunal inventory (45, 46). Several recent papers probably reused overlapping sampling periods, hosts, or localities; they were therefore retained as source-linked records but not pooled as independent specimen totals (43, 47, 48). Sequence accessions and voucher identifiers were inconsistently reported, and checklist decisions remain conditional on the current global classification (25).

## 5 Conclusion

Kazakhstan’s tick fauna is larger, more heterogeneous, and less uniformly confirmed than any single inherited total suggests. Reconciliation of 59 source-name entries supports a strict checklist of 23 verified species and a broader evidence-supported set of 42 species in 10 genera and two families, while 10 additional currently valid taxa are reported separately as requiring validation or taxonomic reassessment. Molecular identification of the tick itself was available for 13 verified species and for 14 of the 42 retained species overall. By linking every decision to a source, locality, specimen context, identification method, and current name, this review provides a documented national baseline. The decisive next step is coordinated voucher-based resampling of unresolved taxa and undersampled regions. Rather than providing another single estimate of tick diversity, this study provides a framework that can be tested, corrected, and used for taxonomy, biodiversity research, veterinary surveillance, and One Health preparedness across Central Asia.
