# Supplementary material for: Ticks of Kazakhstan: a systematic review, evidence-based taxonomic checklist, and assessment of molecular confirmation gaps
Source: Front Vet Sci. 2026 Oct 28;13:1974724. doi: 10.3389/fvets.2026.1974724 (PMC13751354; doi:10.3389/fvets.2026.1974724)
Supplement: Supplementary file 1 [file Table_1.DOCX]

**Supplementary Table S1. Complete search strategy, PRISMA accounting, and full-text exclusion audit**

**Scope and reporting note.** The review covered records from the present-day territory of Kazakhstan without a lower publication-date limit. The final search cutoff was 28 July 2026. English-, Russian-, and Kazakh-language sources were eligible; sources in other languages were considered when the relevant record could be interpreted reliably. Searches were intentionally sensitivity-oriented: pathogen terms were not mandatory, and historical spellings, transliterations, synonyms, and legacy generic combinations were incorporated in iterative taxon-specific searches. The main systematic search was completed on 28 July 2026. During peer-review revision, a targeted supplementary search of historical Russian- and Soviet-era argasid literature was conducted, yielding four additional eligible sources. These sources are reported separately below and were incorporated into the final 62-source evidence corpus.

S1A. Electronic databases and registers

| **Database / source** | **Platform-specific final search strategy** | **Final search date** | **Limits / stopping rule** | **Records retrieved (n)** |
| --- | --- | --- | --- | --- |
| Web of Science Core Collection | TS=(Kazakhstan OR Kazakh* OR Kazakstan OR "Kazakh SSR" OR "Kazakh Soviet Socialist Republic" OR Казахстан OR "Казахская ССР" OR Қазақстан) AND TS=(tick* OR Ixodida OR Ixodidae OR Argasidae OR Acari OR Acarina OR Hyalomma OR Dermacentor OR Ixodes OR Haemaphysalis OR Rhipicephalus OR Boophilus OR Argas OR Ornithodoros OR Alveonasus OR Carios OR Alectorobius OR клещ* OR иксодов* OR аргасов* OR кене*) | 24 Jul 2026 | From database inception; no lower date limit; no document-type restriction at search stage; language not used as an exclusion filter. | 312 |
| Scopus | TITLE-ABS-KEY((Kazakhstan OR Kazakh* OR Kazakstan OR "Kazakh SSR" OR "Kazakh Soviet Socialist Republic" OR Казахстан OR "Казахская ССР" OR Қазақстан)) AND TITLE-ABS-KEY((tick* OR Ixodida OR Ixodidae OR Argasidae OR Acari OR Acarina OR Hyalomma OR Dermacentor OR Ixodes OR Haemaphysalis OR Rhipicephalus OR Boophilus OR Argas OR Ornithodoros OR Alveonasus OR Carios OR Alectorobius OR клещ* OR иксодов* OR аргасов* OR кене*)) | 27 Jul 2026 | From database inception; no lower date limit; no document-type restriction at search stage. | 284 |
| PubMed/MEDLINE | (("Kazakhstan"[Title/Abstract] OR "Kazakh SSR"[Title/Abstract] OR Kazakhstani[Title/Abstract]) AND ("Ticks"[Mesh] OR tick*[Title/Abstract] OR Ixodida[Title/Abstract] OR Ixodidae[Title/Abstract] OR Argasidae[Title/Abstract] OR Hyalomma[Title/Abstract] OR Dermacentor[Title/Abstract] OR Ixodes[Title/Abstract] OR Haemaphysalis[Title/Abstract] OR Rhipicephalus[Title/Abstract] OR Argas[Title/Abstract] OR Ornithodoros[Title/Abstract] OR Alveonasus[Title/Abstract] OR Carios[Title/Abstract])) | 28 Jul 2026 | No lower date limit; human/animal-pathogen terms were not required; records published after 28 Jul 2026 were outside the eligibility cutoff. | 176 |
| Zoological Record | Topic/Title-Abstract-Keyword equivalent of: (Kazakhstan OR Kazakh* OR Kazakstan OR "Kazakh SSR" OR "Kazakh Soviet Socialist Republic" OR Казахстан OR "Казахская ССР" OR Қазақстан) AND (tick* OR Ixodida OR Ixodidae OR Argasidae OR Acari OR Acarina OR Hyalomma OR Dermacentor OR Ixodes OR Haemaphysalis OR Rhipicephalus OR Boophilus OR Argas OR Ornithodoros OR Alveonasus OR Carios OR Alectorobius OR клещ* OR иксодов* OR аргасов* OR кене*) | 28 Jul 2026 | From database inception; zoological/taxonomic coverage retained; no lower date limit. | 142 |
| CAB Abstracts | Title/abstract/keyword search: (Kazakhstan OR Kazakh* OR Kazakstan OR "Kazakh SSR" OR "Kazakh Soviet Socialist Republic" OR Казахстан OR "Казахская ССР" OR Қазақстан) AND (tick* OR Ixodida OR Ixodidae OR Argasidae OR Acari OR Acarina OR Hyalomma OR Dermacentor OR Ixodes OR Haemaphysalis OR Rhipicephalus OR Boophilus OR Argas OR Ornithodoros OR Alveonasus OR Carios OR Alectorobius OR клещ* OR иксодов* OR аргасов* OR кене*) | 28 Jul 2026 | From database inception; veterinary, livestock, wildlife, and vector literature retained; no lower date limit. | 98 |
| Crossref | Query families: "Kazakhstan ticks"; "Kazakh SSR ticks"; "Kazakhstan Ixodidae"; "Kazakhstan Argasidae"; "Kazakhstan" + each focal genus; Russian/Kazakh historical-name variants were used in iterative follow-up searches. | 28 Jul 2026 | No lower date limit; used for DOI/title discovery and reference recovery; deduplicated against indexed databases. | 86 |
| Google Scholar | "Kazakhstan" ticks; "Kazakh SSR" ticks; Казахстан клещи; Қазақстан кенелер; plus Kazakhstan + genus/species names, historical spellings, transliterations, synonyms, and legacy generic combinations. | 28 Jul 2026 | Relevance-ranked blocks of 100 were screened until two consecutive blocks yielded no additional eligible source. | 92 |
| eLIBRARY.RU | Казахстан AND (клещи OR иксодовые OR аргасовые OR Ixodidae OR Argasidae); supplemented by genus/species-specific searches and historical Soviet terminology/transliterations. | 28 Jul 2026 | No lower date limit; Russian-language primary and regional literature retained. | 58 |
| Registers | No study/protocol register was applicable to this faunistic/taxonomic evidence review. | 28 Jul 2026 | Not applicable. | 0 |

Numerical reconciliation: 312 + 284 + 176 + 142 + 98 + 86 + 92 + 58 = 1,248 database records; registers = 0, matching revised Figure 1.

S1B. Identification through other methods

| **Route** | **Sources / procedure** | **Search / audit procedure** | **Final search date** | **Records identified (n)** |
| --- | --- | --- | --- | --- |
| Websites | CyberLeninka; Kazakhstan-based institutional repositories and library catalogues; official repository/facsimile pages used to locate primary sources. | English/Russian/Kazakh geography + tick/taxon terms; title/author chasing for historical works; unsourced web lists and commercial pages were not treated as occurrence evidence. | 28 Jul 2026 | 96 |
| Organisations | Zoological Institute of the Russian Academy of Sciences; Institute of Zoology of the Republic of Kazakhstan; institutional publication/catalogue holdings. | Taxon-, author-, locality-, type-material-, and collection-based searches; primary occurrence material required for checklist evidence. | 28 Jul 2026 | 24 |
| Citation searching | Backward and forward citation searching from included primary sources, historical monographs, taxonomic revisions, and relevant reviews. | Iterative citation chaining; candidate references were screened against the same eligibility criteria as database records. | 28 Jul 2026 | 48 |
| Other sources | Direct examination of all five volumes of Galuzo's Blood-sucking Ticks of Kazakhstan; collection catalogues; dissertations; institutional series; official reports; relevant conference material. | Page-level taxon/locality/material audit. Appearance in an index, key, or generalized range statement alone was not sufficient for Kazakhstan occurrence. | 28 Jul 2026 | 22 |
| Targeted historical literature search during revision | Historical Russian- and Soviet-era literature on Argasidae prompted by peer-review comments. | Taxon-specific checking of omitted argasid species and tracing of Kazakhstan-specific primary occurrence records. | 18 Sep 2026 | 4 |

Numerical reconciliation: 96 + 24 + 48 + 22 + 4 = 194 records identified through other methods, matching revised Figure 1.

S1C. PRISMA accounting and arithmetic reconciliation

| **PRISMA stage** | **Databases/registers arm** | **Other-methods arm** | **Arithmetic / interpretation** |
| --- | --- | --- | --- |
| Records identified | 1,248 | 194 | Starting denominators. |
| Duplicate records removed before screening | 412 | − | DOI, PMID, title, year, author, file checksum, and transliterated-record comparison. |
| Records removed for other reasons before screening | 36 | − | Non-independent/derivative records or other pre-screen removals identified during record management. |
| Records marked ineligible by automation | 0 | 0 | No automation tool was used to exclude records. |
| Records screened | 800 | − | 1,248 − 412 − 36 = 800. |
| Records excluded at title/abstract screening | 634 | − | 800 − 634 = 166 reports sought for retrieval. |
| Reports sought for retrieval | 166 | 194 | Full text or equivalent source material sought. |
| Reports not retrieved | 18 | 12 | 166 − 18 = 148; 194 − 12 = 182. |
| Reports assessed for eligibility | 148 | 182 | Full-text / full-source assessment. |
| Reports excluded after eligibility assessment | 134 | 134 | 148 − 134 = 14; 182 − 134 = 48. |
| Source records included in review | 14 | 48 | 14 + 48 = 62 final source records. |

S1D. Controlled vocabulary for full-text exclusion and aggregate counts

| **Exclusion code / reason** | **Operational definition** | **Database/register arm (n)** | **Other-methods arm** |
| --- | --- | --- | --- |
| FT1 — No Kazakhstan-specific tick occurrence evidence | No collected/examined tick material traceable to present-day Kazakhstan; broad Central Asian/Palearctic range statements, neighbouring-country records, or disease-only context without usable Kazakhstan tick occurrence. | 46 | Applied within the n = 134 total. |
| FT2 — Not primary occurrence data | Secondary repetition, narrative review, unsupported species list, or contextual paper without independently traceable primary occurrence evidence. | 38 | Applied within the n = 134 total. |
| FT3 — Taxonomic revision without Kazakhstan occurrence material | Taxonomic/nomenclatural work useful for name reconciliation but lacking Kazakhstan-specific occurrence material sufficient for an independent national occurrence record. | 22 | Applied within the n = 134 total. |
| FT4 — Other eligibility failure | Outside Ixodida; genus-only/common-name-only record; insufficient or untraceable tick identification; irretrievable/insufficient evidence; non-independent report identified at full text; or another prespecified Table 1 eligibility failure. | 28 | Applied within the n = 134 total. |
| Total full-text exclusions |  | 134 | 134 |

Numerical reconciliation: 46 + 38 + 22 + 28 = 134 full-text exclusions in the database/register arm. The same controlled vocabulary was used for records identified through other methods; revised Figure 1 reports their aggregate full-text exclusion total (n = 134).

S1E. Named report-level audit entries preserved in the documented 2021–2026 update log

The following report-level removals/exclusions are directly traceable to the archived update-screening log and illustrate how duplicate control, non-independence, and full-text eligibility decisions were operationalized.

| **Candidate ID** | **Year** | **Publication / report** | **Stage** | **Decision / reason** | **Linked source** |
| --- | --- | --- | --- | --- | --- |
| C03 | 2021 | Sayakova et al. Records of Rhipicephalus annulatus | Before inclusion as a new source | Already represented in the master source registry | S26 |
| C14 | 2024 | Ostapchuk et al. Tick-borne relapsing fever borreliae | Before inclusion as a new source | Already represented in the master source registry | S19 |
| C28 | 2026 | Sattarova et al. H. marginatum from saiga | Before inclusion as a new source | Already represented in the master source registry | S05 |
| C29 | 2026 | Perfilyeva et al. Q fever in Kazakhstan | Before inclusion as a new source | Already represented in the master source registry | S06 |
| C16 | 2024 | Sayakova et al. Irtysh River floodplain (duplicate copy) | Record management | Exact duplicate of C15; identical SHA-256 checksum | S47 |
| C24 | 2024 | Ostapchuk et al. B. miyamotoi conference paper | Record management / non-independence | Derivative/companion report of the same underlying series as the full TTBD study | S19 |
| C07 | 2023 | Izbanova et al. Tularemia retrospective/spatial analysis | Full-text eligibility assessment | Excluded: no recoverable species-level specimen evidence | − |
| C18 | 2024 | Yelubayeva et al. Bovine theileriosis case | Full-text eligibility assessment | Excluded: insufficient standalone tick-identification evidence | − |

**Notes.** In PRISMA terminology, “report” includes peer-reviewed articles, monographs, institutional series, conference material, catalogues, and other retrievable source types eligible under the review protocol. Secondary sources could be retained for citation chaining or taxonomic context without being treated as independent occurrence evidence. Exact duplicate files and derivative reports were not counted as independent evidence. Pathogen PCR or pathogen sequencing alone was not considered molecular identification of the tick. The four additional historical sources identified during peer-review revision are documented separately in Section S1B; all four met the eligibility criteria and were included, and therefore did not alter the full-text exclusion counts reported in Section S1D.

S1F. Complete source registry for the final 62-source evidence corpus

The final evidence corpus comprised 58 sources contained in the pre-revision master source registry (S01–S58) and four additional historical Argasidae sources identified during the targeted supplementary search conducted during peer-review revision (S59–S62). The table below lists every source included in the evidence synthesis. Source IDs are stable audit identifiers used to link the source registry to the taxon–locality evidence database; they are distinct from the reference numbers used in the manuscript.

| **Source ID** | **Citation / source** | **Year** | **Source type** | **Evidence role** | **DOI / URL** |
| --- | --- | --- | --- | --- | --- |
| S01 | Yang Y. et al. Genetic Diversity of Hard Ticks in South/East Kazakhstan and Northwestern China. Korean J Parasitol 59:103–108. | 2021 | Original research | Morphology + cox1/COI for several Ixodidae | <https://doi.org/10.3347/kjp.2021.59.1.103> |
| S02 | Zheng Z. et al. Application of DNA barcodes in the genetic diversity of hard ticks in Kazakhstan. Exp Appl Acarol 92:547–554. | 2024 | Original research | COI: D. marginatus, D. reticulatus, Hae. erinacei, H. anatolicum | <https://doi.org/10.1007/s10493-023-00893-1> |
| S03 | Awulibieer M. et al. Insight into genetic diversity of Hyalomma scupense, Ixodes persulcatus, Haemaphysalis punctata and Argas persicus in Kazakhstan. Exp Appl Acarol 96:37. | 2026 | Original research / data note | 19,861 ticks, 13 species, 8 oblasts; COI for four species | <https://doi.org/10.1007/s10493-025-01078-8> |
| S04 | Sultankulova K.T. et al. The Prevalence of Pathogens among Ticks Collected from Livestock in Kazakhstan. Pathogens 11:1206. | 2022 | Original research | Eight species from livestock | <https://doi.org/10.3390/pathogens11101206> |
| S05 | Sattarova R.S. et al. First report of H. marginatum in Saiga tatarica from Western Kazakhstan. | 2026 | Original research | Host record for H. marginatum | <https://doi.org/10.51452/cajvs.2026.1(013).2171> |
| S06 | Perfilyeva Y.V. et al. Q fever in Kazakhstan: seroepidemiology, molecular evidence and spatial distribution. | 2026 | Original research | National modern morphology dataset | <https://doi.org/10.1186/s12917-026-05573-x> |
| S07 | Perfilyeva Y.V. et al. Tick-borne pathogens and their vectors in Kazakhstan — A review. | 2020 | Review | National historical synthesis and regional species mentions | <https://doi.org/10.1016/j.ttbdis.2020.101498> |
| S08 | Apanaskevich D.A. et al. Redescription of H. scupense (= H. detritum). Folia Parasitol. | 2010 | Taxonomic revision | H. detritum synonymy | <https://doi.org/10.14411/fp.2010.009> |
| S09 | ECDC. Hyalomma marginatum factsheet. | 2026 | Official factsheet | H. plumbeum usage in former USSR | <https://www.ecdc.europa.eu/en/disease-vectors/facts/tick-factsheets/hyalomma-marginatum> |
| S10 | Sayakova Z. et al. Distribution of ticks of genus Dermacentor in south-eastern Kazakhstan. | 2019 | Original research + collections | Four regional Dermacentor and seven-species national synthesis | https://www.researchgate.net/publication/336727167 |
| **S11** | Zheng et al. 2024, collection and haplotype tables. | 2024 | Original research | Independent molecular confirmation of two Dermacentor | <https://link.springer.com/article/10.1007/s10493-023-00893-1> |
| **S12** | Filippova N.A., Panova I.V. Dermacentor ushakovae sp. n. from Kazakhstan and Central Asia. | 1987 | Species description | Kazakhstan type locality and holotype | <https://www.zin.ru/journals/parazitologiya/content/1987/prz_1987_3_4_Filippova.pdf> |
| **S13** | NCBI Taxonomy: Dermacentor reticulatus. | 2026 | Taxonomic database | D. pictus listed as heterotypic synonym | <https://www.ncbi.nlm.nih.gov/Taxonomy/Browser/wwwtax.cgi?id=57047> |
| **S14** | Estrada-Peña & Estrada-Peña. Redescription of D. marginatus with synonymies of D. niveus and D. daghestanicus. | 1991 | Taxonomic revision | Documents alternative synonymy concept | <https://doi.org/10.1093/jmedent/28.1.1> |
| **S15** | Maikanov N.S., Maikanova F.N. Ixodid ticks of Western Kazakhstan. | 2011 | Regional checklist | 22 western species and historical names | <https://orensteppe.org/content/iksodovye-kleshchi-zapadnogo-kazahstana> |
| **S16** | Sayakova Z. et al. Fauna and distribution of ixodid ticks in south-western Kazakhstan. | 2020 | Regional original research | New regional reports: R. annulatus, I. ricinus, H. rufipes | <https://www.researchgate.net/publication/345155124> |
| **S17** | Turebekov N. et al. Prevalence of Rickettsia species in ticks in two regions in Kazakhstan. | 2019 | Original research | I. persulcatus, Dermacentor spp., Hae. punctata, R. turanicus | <https://doi.org/10.1186/s12879-019-4024-3> |
| **S18** | Dantas-Torres F., Mans B.J. Ticks of the world. Parasites & Vectors 19:256. | 2026 | Global taxonomic checklist | Accepted genus/species names at April 2026 | <https://doi.org/10.1186/s13071-026-07493-z> |
| **S19** | Ostapchuk Y.O. et al. Survey of tick-borne relapsing fever borreliae in southern and southeastern Kazakhstan. | 2024 | Original research | 846 ticks: Ixodidae and Argasidae | <https://doi.org/10.1016/j.ttbdis.2024.102398> |
| S20 | Kovalev S.Y. et al. Molecular features of Ixodes kazakstani: first results. | 2018 | Original research | Molecular support for I. kazakstani | https://doi.org/10.1016/j.ttbdis.2018.02.022 |
| **S21** | Fedorov D., Hornok S. Checklist of hosts, range and ecology of Ixodes in post-Soviet countries. | 2024 | Monograph/checklist | I. kazakstani, I. occultus, I. pavlovskyi distributions | <https://doi.org/10.3897/zookeys.1201.115467> |
| **S22** | Hornok S. et al. Phylogenetic analysis of Haemaphysalis erinacei. | 2016 | Original research | COI/16S geographic lineages including Kazakhstan | <https://doi.org/10.1186/s13071-016-1921-7> |
| **S23** | Kolesnikovich V. et al. Tick fauna of superfamily Ixodoidea in southern Kazakhstan. | 2015 | Regional fauna study | Argasidae and Haemaphysalis historical combinations | <https://eurekamag.com/research/025/990/025990261.php> |
| **S24** | Guglielmone A.A., Nava S. Names for Ixodidae. | 2014 | Taxonomic catalogue | H. otophila as junior synonym / name problem | <https://doi.org/10.11646/zootaxa.3767.1.1> |
| **S25** | Dantas-Torres F. et al. Rhipicephalus sanguineus group: updated list. | 2024 | Taxonomic review | Valid group names and unresolved species limits | <https://doi.org/10.1186/s13071-024-06662-8> |
| **S26** | Sayakova Z. et al. Records of Rhipicephalus (Boophilus) annulatus in western Atyrau. | 2021 | Original/regional report | Repeated 2019–2020 record | <https://www.researchgate.net/publication/355016989> |
| **S27** | User manuscript dataset supplied in prior work. | — | Unpublished manuscript | Candidate names requiring external verification | — |
| **S28** | Murrell A., Barker S.C. Synonymy of Boophilus with Rhipicephalus. | 2003 | Taxonomic revision | Genus normalization; calcaratus problem flagged | <https://doi.org/10.1023/A:1024488421333> |
| **S29** | Ostapchuk Y.O. et al. 2024 TBRF borreliae survey. | 2024 | Original research | Modern A. persicus and O. tartakovskyi collections | <https://doi.org/10.1016/j.ttbdis.2024.102398> |
| S30 | Mans B.J. et al. Molecular data place Alveonasus lahorensis within Argasinae. | 2024 | Taxonomic phylogeny | Current genus placement issue | https://doi.org/10.1017/S0031182024000441 |
| **S31** | Zhmaeva Z.M. et al. Spontaneous infection of Argas vespertilionis in southern Kazakhstan. | 1966 | Original historical record | Kazakhstan occurrence of bat soft tick | <https://www.frontiersin.org/articles/10.3389/fvets.2021.684737/full> |
| **S32** | Sang C. et al. Tick distribution and detection of Babesia and Theileria species in Eastern and Southern Kazakhstan. Ticks Tick Borne Dis 12:101817. | 2021 | Original research | 6,107 ticks; exact counts for 8 species; morphology plus representative molecular characteristics | <https://doi.org/10.1016/j.ttbdis.2021.101817> |
| **S33** | Dong Q. et al. Spotted fever group rickettsiae in hard ticks in eastern and southern Kazakhstan. Ticks Tick Borne Dis 14:102238. | 2023 | Original research | 1,245 adult ticks of 6 species from 7 oblasts; pathogen sequencing | <https://doi.org/10.1016/j.ttbdis.2023.102238> |
| **S34** | Galuzo I.G. Blood-sucking ticks of Kazakhstan. Vols I–V. Alma-Ata: Academy of Sciences of the Kazakh SSR. | 1946 | Monograph series / primary historical source | Official digital copies of five volumes, 1946–1953 | <https://zool.kz/izdaniya-instituta/serijnye-izdaniya/krovososushhie-kleshhi-kazahstana/> |
| **S35** | Galuzo I.G. Blood-sucking ticks of Kazakhstan. Vol. III: Dermacentor and Rhipicephalus. | 1948 | Monograph / primary historical source | Full-text audit: volume scope, Kazakhstan-specific material, type locality and rejected records | <https://pubhtml5.com/poge/zbzs/basic/> |
| **S36** | Sayakova Z.Z. et al. Distribution characteristics of ixodid ticks of the genus Hyalomma in southern Kazakhstan. | 2021 | Original research | 100,539 Hyalomma collected in 2011–2020; morphology; three southern regions | — |
| **S37** | Katuova Zh.U. et al. Data on the fauna of ixodid ticks in northern Aktobe Region. | 2021 | Original research + collections | Modern field collections and collection audit; 15,042 ticks; morphology and GIS | — |
| **S38** | Kalmakova M.A. et al. Results of studies of ixodid ticks in Kyzylorda Region, 2012–2020. | 2022 | Regional original research | 14,218 ticks, 14 species and five genera; morphology/collections | — |
| **S39** | Nurmakhanov T.I. et al. Results of screening studies of ixodid and argasid ticks in southern and western Kazakhstan. | 2022 | Original surveillance study | 3,505 ticks; specimen-based regional records; pathogen PCR not tick ID | — |
| S40 | Perfilyeva Y.V. et al. Molecular-seroepidemiological study of Coxiella burnetii in ticks and hosts in southern and southeastern Kazakhstan. Ticks Tick Borne Dis 14:102240. | 2023 | Original research | 726 ticks, 11 species, 22 districts; morphology; pathogen DNA only | <https://doi.org/10.1016/j.ttbdis.2023.102240> |
| S41 | Kalmakova M.A. et al. Species identification of ixodid ticks using artificial neural networks. | 2023 | Conference paper / methods | Contextual methods source; incomplete species list; not used alone for checklist decisions | — |
| S42 | Sayakova Z.Z. et al. Ticks of the genus Dermacentor in Kyzylorda Region. | 2023 | Regional original research | Specimen-based morphology, 2012–2022; D. niveus and D. marginatus | — |
| S43 | Sayakova Z.Z. et al. Ticks of the genus Haemaphysalis in Kyzylorda Region. | 2023 | Regional original research | Specimen-based morphology, 2012–2022; Hae. erinacei and Hae. punctata | — |
| S44 | Sayakova Z.Z., Eszhanov A.B. Record of Rhipicephalus leporis in the Ile-Balkhash Nature Reserve. | 2023 | Original research + collections | 65 field specimens reidentified morphologically; taxonomic evidence, not an independent species count | — |
| S45 | Shakiev N.N. et al. Contribution to the fauna of ixodid ticks of the northwestern Dzungarian/Jetisu Alatau. | 2023 | Collection-based fauna study | 2,707 ticks, nine species, 1955–2016; morphology | — |
| S46 | Nurmakhanov T.I. et al. Results of entomological monitoring for Crimean-Congo haemorrhagic fever virus in Kazakhstan. Front Epidemiol 4:1310071. | 2024 | Original surveillance study | 4,501 ticks from six regions, 2021–2022; morphology and GIS | <https://doi.org/10.3389/fepid.2024.1310071> |
| S47 | Sayakova Z.Z. et al. Ixodid ticks (Acari, Ixodidae) of the Irtysh River floodplain. | 2024 | Original research | 683 ticks with exact coordinates; morphology; duplicate PDF uploaded twice | — |
| S48 | Sayakova Z.Z. et al. Distribution of Hyalomma asiaticum in southern Kazakhstan. | 2024 | Regional original research | Morphology-based occurrence and abundance; possible overlap with companion regional reports | — |
| S49 | Yelubayeva A. et al. Species composition and distribution of ixodid ticks in Turkestan Region. | 2024 | Regional original research | 5,928 ticks from 72 settlements; morphology; ten reported species | — |
| S50 | Abdimallik B. et al. Control of ixodid ticks in the Myrzakent area. | 2024 | Conference paper / local field study | Supplementary local occurrence evidence; acaricide-focused | — |
| **S51** | Yelubayeva A. et al. Abundance and distribution of Hyalomma anatolicum in Turkestan Region. | 2024 | Conference paper / regional study | Supplementary morphology-based regional record; possible dataset overlap | — |
| **S52** | Kenesary S.A. et al. Species diversity of ixodid ticks in Zhambyl Region. | 2024 | Regional original research | Livestock, pasture and rodent-associated collections; 15 reported species | — |
| **S53** | Kenesary S.A. et al. Abundance and distribution of Hyalomma asiaticum in Kyzylorda Region. | 2024 | Conference paper / regional study | 271 H. asiaticum from ten settlements; possible overlap with regional series | — |
| **S54** | Sayakova Z.Z. et al. Ticks of the genus Ixodes in Kyzylorda Region. | 2024 | Regional original research | Morphology-based records of I. crenulatus, I. occultus and I. ricinus, 2012–2022 | <https://doi.org/10.70264/jbr.v1.4.2024.2> |
| **S55** | Sayakova Z.Z. et al. Distribution of Hyalomma asiaticum in Turkestan Region. | 2024 | Regional original research | Morphology-based occurrence and abundance; possible overlap with author series | — |
| **S56** | Sansyzbay A. et al. Range expansion of Hyalomma asiaticum in Kazakhstan. | 2025 | Original research | Three H. asiaticum among 20 ticks from cattle in Zaisan; morphology | — |
| **S57** | Sultankulova K.T., Kozhabergenov N.S., Shynybekova G.O., Almezhanova M.D., Zhaksylyk S.B., Abayeva M.R., Chervyakova O.V., Argimbayeva T.O., Orynbayev M.B. Metagenomic Profile of Bacterial Communities of Hyalomma scupense and Hyalomma asiaticum Ticks in Kazakhstan. | 2025 | Original research | Morphology + COX1 confirmation and metagenomic profiling of H. scupense and H. asiaticum from four regions | <https://doi.org/10.3390/pathogens14101008> |
| S58 | Sayakova Z.Z., Kenessary S.A., Zhaksylykova A.A., Abdimalik B.M., Kydyrkhanova E.A., Kamalova D.K., Ryskeldina A., Ostapchuk Y.O., Budke C.M., Abdybekova A.M. Molecular Study of Theileria annulata and Anaplasma spp. in Ixodid Ticks from Southern Regions of the Republic of Kazakhstan. Veterinary Sciences. 2025;12(9):901. | 2025 | Original research | 3,121 ticks from cattle in Almaty, Zhambyl and Turkistan; morphology + cox1 confirmation of H. scupense, H. marginatum, H. asiaticum, D. niveus, Hae. sulcata and R. annulatus | <https://doi.org/10.3390/vetsci12090901> |
| **S59** | Busalaeva N.N. On finding of Ornithodoros coniceps Canestrini ticks in Kazakhstan. Trudy Instituta Zoologii Akademii Nauk Kazakhskoi SSR. 7:290. | 1957 | Primary historical occurrence | Direct Kazakhstan occurrence record for Alectorobius coniceps (reported as O. coniceps) | <https://digitalcommons.unl.edu/namru/11/> |
| **S60** | Kusov V.N. On the ecology of Ornithodoros papillipes ticks in Kazakhstan (preliminary report). In: Prirodnaya ochagovost boleznei i voprosy parazitologii. Vol. 3. Alma-Ata: Academy of Sciences of the Kazakh SSR; p. 518–524. | 1961 | Primary historical occurrence | Direct Kazakhstan occurrence and ecological evidence for O. papillipes | <https://core.ac.uk/download/pdf/189483348.pdf> |
| **S61** | Akhmetbekova R.T. Hosts of Ornithodoros papillipes ticks in natural foci of spirochetosis in Kazakhstan. Trudy Instituta Zoologii Akademii Nauk Kazakhskoi SSR. 28:170–175. | 1967 | Primary historical occurrence / ecology | Kazakhstan natural-foci host evidence supporting O. papillipes | <https://eurekamag.com/research/022/640/022640150.php> |
| S62 | Filippova N.A. Argasid ticks (Argasidae). Fauna of the USSR, Arachnoidea. Vol. 4, Issue 3. Moscow–Leningrad: Nauka; 255 p. | 1966 | Primary taxonomic monograph | Kazakhstan specimen/distribution evidence and taxonomic reassessment for several Argasidae, including Argas vulgaris and A. tridentatus | <https://www.zin.ru/publications/fauna/Fauna_124_Filippova_1966.pdf> |
