# Supplementary material for: Ticks of Kazakhstan: a systematic review, evidence-based taxonomic checklist, and assessment of molecular confirmation gaps
Source: Front Vet Sci. 2026 Oct 28;13:1974724. doi: 10.3389/fvets.2026.1974724 (PMC13751354; doi:10.3389/fvets.2026.1974724)
Supplement: Supplementary file 2 [file Table_2.docx]

# Supplementary

**Table S2. Species-level evidence audit and source-name reconciliation supporting the national checklist of ticks reported from Kazakhstan.**

The table contains all 59 source-name entries. The same accepted species may therefore appear in more than one row. Reference numbers correspond to the manuscript reference list. Molecular confirmation denotes molecular identification of the tick itself, not detection of a pathogen. Source-reported names are retained verbatim to preserve auditability.

| Source name | Accepted name (2026) | Taxonomic action | Kazakhstan evidence / region | Identification method | Tick molecular ID | Evidence category | Checklist decision | Principal source(s) | Taxonomic rationale / critical note |
| --- | --- | --- | --- | --- | --- | --- | --- | --- | --- |
| *Hyalomma asiaticum* | *Hyalomma asiaticum* | accepted | Southern, south-eastern, eastern and western Kazakhstan; repeated modern collections, including locality-linked *COI/cox1*-confirmed records from Zhambyl and Jetisu regions, with additional modern records from Almaty and Turkistan. | Morphology + *COI/cox1* | Yes | Molecularly confirmed | Include | [20, 22, 23, 28, 34] | Subspecific combinations were collapsed and not counted separately. |
| *Hyalomma anatolicum / H. a. anatolicum* | *Hyalomma anatolicum* | accepted; collapse subspecies | Zhambyl, Turkestan and other southern regions. | Morphology + *COI* | Yes | Molecularly confirmed | Include | [39, 40] | Potential confusion with *H. excavatum* requires continued taxonomic control. |
| *Hyalomma scupense* | *Hyalomma scupense* | accepted | Eight regions and 41 sites in the 2016–2024 dataset, with additional locality-linked *COI/cox1*-confirmed records from Kyzylorda, Zhambyl and Turkistan regions. | Morphology + *COI/cox1* | Yes | Molecularly confirmed | Include | [13, 20, 23, 38, 39] | Well-supported occurrence; *H. detritum* records reconcile to this species. |
| *Hyalomma marginatum* | *Hyalomma marginatum* | accepted | Western and southern Kazakhstan; recent field records, including *Saiga tatarica*, with Kazakhstan *cox1*-confirmed material from Zhambyl and Turkistan regions. | Morphology + *COI/cox1* | Yes | Molecularly confirmed | Include | [21, 23, 41] | Historical *H. plumbeum* records require source-level reconciliation; the available Kazakhstan cox1 evidence is based on a limited number of sequenced specimens. |
| *Hyalomma excavatum* | *Hyalomma excavatum* | accepted | Eight regions; 325 specimens in the 2016–2024 dataset. | Morphology | No | Verified morphology | Include Provisional | [37, 38] | COI confirmation is a priority because of similarity to *H. anatolicum.* |
| *Hyalomma dromedarii / dromedaryi* | *Hyalomma dromedarii* | spelling normalization | Mangystau Region; 49 morphologically identified specimens collected in 2021–2022. | Morphology using taxonomic keys | No | Verified morphology | Include | [37] | Modern specimen-based evidence resolves the former primary-evidence gap; no tick sequence. |
| *Hyalomma rufipes* | *Hyalomma rufipes* | accepted globally | Rare modern records from southern Kazakhstan, potentially associated with dispersal. | Morphology | No | New/possible record | Include Provisional | [42] | Current evidence cannot distinguish establishment from introduction; voucher and sequence needed. |
| *Hyalomma turanicum* | *Hyalomma turanicum* | accepted globally | Repeated modern records from Kyzylorda, Turkestan and Zhambyl regions. | Morphology | No | Verified morphology / taxonomic caution | Include Provisional | [39] | Presence is supported morphologically, but the difficult *Hyalomma* complex needs voucher-based sequencing. |
| *Hyalomma detritum* | *Hyalomma scupense* | synonymize | Historical and modern records from southern and eastern Kazakhstan. | Morphology + cox1 | Yes; reported under a legacy name | Synonymized | Do Not Count Separately | [14, 21, 24, 38, 39] | Provides evidence for *H. scupens*e and is not counted as a separate species. |
| *Hyalomma plumbeum* | *Hyalomma marginatum* | obsolete regional usage | Historical records from southern Kazakhstan. | Morphology using legacy keys | No | Synonymized/questionable | Do Not Count Separately | [21, 23, 41] | Legacy regional usage; retained for traceability but not counted independently. |
| *Dermacentor marginatus* | *Dermacentor marginatus* | accepted | Almaty, Jetisu, Zhambyl, Turkestan and western regions. | Morphology + *COI* | Yes | Molecularly confirmed | Include | [19, 40] | Supported by large collections and Kazakhstan COI haplotypes. |
| *Dermacentor reticulatus* | *Dermacentor reticulatus* | accepted | Almaty, southern and other regions. | Morphology + *COI* | Yes | Molecularly confirmed | Include | [19, 40] | Historical *D. pictus* records reconcile to this species. |
| *Dermacentor niveus* | *Dermacentor niveus* | accepted in 2026 list; disputed complex | Southern and south-eastern Kazakhstan; historical and modern records, including *cox1*-confirmed material from Almaty Region. | Morphology + *COI/cox1* | Yes | Molecularly confirmed | Include | [19,23,37] | Kazakhstan *cox1* evidence supports strict inclusion, but boundaries within the *D. niveus-D. marginatus* complex still require integrative revision. |
| *Dermacentor pavlovskyi* | *Dermacentor pavlovskyi* | accepted | Type locality: Ak-Kenil tract, Karatau Range, former Syr-Darya Region, now Kazakhstan. | Type series + morphology/collections | No | Type-locality verified | Include | [15] | Type series: two males and one female collected on 31 January 1910; museum no. 43–1910. |
| *Dermacentor ushakovae* | *Dermacentor ushakovae* | accepted | Type locality: Chilik River valley, Kazakhstan. | Original type description | No | Type-locality verified | Include | [57] | Holotype held at ZIN; present distribution requires reassessment. |
| *Dermacentor nuttalli* | *Dermacentor nuttalli* | accepted globally | Black Irtysh floodplain and Pavlodar Region; 22 specimens collected in 2023. | Morphology using taxonomic keys | No | Verified morphology | Include | [35] | Modern georeferenced specimen evidence supports strict inclusion. |
| *Dermacentor silvarum* | *Dermacentor silvarum* | accepted globally | Listed nationally in 2019; the underlying primary Kazakhstan record was not recovered. | Secondary checklist | No | Historical supported | Pending Primary | [19] | Not accepted as verified until a collection or primary locality record is traced. |
| *Dermacentor pictus* | *Dermacentor reticulatus* | heterotypic synonym | Frequent in historical regional tables. | Morphology using historical keys | No | Synonymized | Do Not Count Separately | [19, 40] | Heterotypic synonym of *D. reticulatus*; not counted separately. |
| *Dermacentor daghestanicus* | *Dermacentor niveus / D. marginatus complex* | name absent from 2026 valid list; disputed synonymy | Galuzo reported multiple western and eastern Kazakhstan collections. | Historical morphology/material summary | No | Historical primary / disputed taxon | Do Not Count Separately | [15, 26, 46] | Primary occurrence evidence exists, but the source name is not counted because synonymy remains conflicting. |
| *Dermacentor raskemensis* | *Dermacentor raskemensis* | accepted globally | Mentioned by Galuzo, but no Kazakhstan-specific locality was recovered. | Historical mention | No | Questionable | Exclude Until Verified | [17, 26] | Global validity does not establish national occurrence. |
| *D. variegatus kamtschadalus* | *Dermacentor kamshadalus* | old combination / spelling normalized | Historical mention without a Kazakhstan-specific locality. | Historical mention | No | Questionable | Exclude Until Verified | [17, 26] | Normalized to *D. kamshadalus* but excluded from the national checklist pending primary evidence. |
| *Ixodes persulcatus* | *Ixodes persulcatus* | accepted | Almaty and eastern/south-eastern Kazakhstan. | Morphology + *COI* in a Kazakhstan dataset | Yes | Molecularly confirmed | Include | [17, 18, 38] | One of the most consistently supported *Ixodes* records. |
| *Ixodes ricinus* | *Ixodes ricinus* | accepted | Western Kazakhstan and modern collections from animals. | Morphology | No Kazakhstan sequence verified | Verified morphology | Include Provisional | [21, 36, 42] | A Kazakhstan voucher-linked sequence remains necessary. |
| *Ixodes kazakstani* | *Ixodes kazakstani* | accepted | South-eastern Kazakhstan; relict disjunct distribution. | Morphology; 16S/ITS2 data outside Kazakhstan | No Kazakhstan sequence verified | Verified morphology | Include | [16] | Accepted as a distinct species, but published 16S/ITS2 sequences were from Kyrgyzstan, not Kazakhstan. |
| *Ixodes occultus* | *Ixodes occultus* | accepted | Kyzylorda Region; great gerbils and burrows in several districts, 2012–2022. | Morphology using taxonomic keys | No | Verified morphology | Include | [36] | Modern specimen-based records provide host/substrate and locality evidence. |
| *Ixodes crenulatus* | *Ixodes crenulatus* | accepted | Zhanakorgan District and Kyzylorda city; specimens from a weasel and fox, 2012–2022. | Morphology using taxonomic keys | No | Verified morphology | Include | [36] | Modern specimen-based records provide host and locality evidence. |
| *Ixodes laguri* | *Ixodes laguri* | accepted | West Kazakhstan, Atyrau, Aktobe and Pavlodar regions. | Morphology/regional records | No | Historical supported | Include Provisional | [37, 43] | Historical sources may use the combination *I. laguri laguri*. |
| *Ixodes redikorzevi* | *Ixodes redikorzevi* | accepted | West Kazakhstan and Atyrau regions. | Morphology/regional record | No | Historical supported | Pending Primary | [43] | A primary voucher and modern identification check are still required. |
| *Ixodes lividus* | *Ixodes lividus* | accepted | Reported from Pavlodar Region. | Morphology/secondary report | No | Historical supported | Pending Primary | [12] | A *nidicolous* bird tick; the primary Kazakhstan locality record remains to be recovered. |
| *Ixodes pavlovskyi* | *Ixodes pavlovskyi* | accepted | Eastern Kazakhstan in a modern post-Soviet checklist. | Literature and collection review | No Kazakhstan sequence verified | Historical supported | Include Provisional | [16] | Morphologically close *to I. persulcatus*; Kazakhstan material requires molecular audit. |
| *Haemaphysalis punctata* | *Haemaphysalis punctata* | accepted | Almaty, Kyzylorda and western regions. | Morphology + *COI* in a Kazakhstan dataset | Yes | Molecularly confirmed | Include | [17, 35, 38] | Supported by both historical and modern records, including Kazakhstan COI data. |
| *Haemaphysalis erinacei* | *Haemaphysalis erinacei* | accepted | Almaty and western/south-eastern Kazakhstan. | Morphology + *COI* | Yes | Molecularly confirmed | Include | [35, 40] | Kazakhstan COI haplotypes have been published. |
| *Haemaphysalis concinna* | *Haemaphysalis concinna* | accepted | Black Irtysh floodplain and Pavlodar Region; 88 specimens collected in 2023. | Morphology using taxonomic keys | No | Verified morphology | Include | [35] | Modern georeferenced series resolves the former primary-evidence gap. |
| *Haemaphysalis numidiana / H. n. turanica* | *Haemaphysalis numidiana complex* | subspecies/taxonomy unresolved in corpus | Western and southern Kazakhstan. | Morphology using regional keys | No | Questionable taxon | Pending Taxonomy | [12, 43] | Do not merge automatically with *H. erinacei* without type and key reassessment. |
| *Haemaphysalis otophila* | *Haemaphysalis parva* | junior synonym in modern treatments | Reported from southern Kazakhstan. | Morphology | No | Synonymized/questionable | Do Not Count Separately | [26] | Modern treatments place the name in synonymy with *H. parva*; specimens require reassessment. |
| *Rhipicephalus turanicus* | *Rhipicephalus turanicus* | accepted | Western, southern and south-eastern Kazakhstan. | Morphology + *COI/cox1* | Yes | Molecularly confirmed | Include | [17, 22, 39] | Member of the *R. sanguineus* group; reference-quality barcodes remain desirable. |
| *Rhipicephalus annulatus / Boophilus annulatus* | *Rhipicephalus annulatus* | genus normalization | Atyrau, Almaty, Zhambyl and Turkistan regions; repeated modern field records, including Kazakhstan cox1-confirmed material. | Morphology + *COI/cox1* | Yes | Molecularly confirmed | Include | [23, 39, 42] | Modern occurrence and Kazakhstan *cox1* data support strict inclusion; broader voucher-linked sampling is still desirable. |
| *Rhipicephalus schulzei* | *Rhipicephalus schulzei* | accepted | West Kazakhstan, Atyrau and Aktobe regions. | Morphology/regional dataset | No | Historical supported | Include Provisional | [37, 43, 45] | Member of the *R. sanguineus* group; modern Kazakhstan barcoding is needed. |
| *Rhipicephalus rossicus* | *Rhipicephalus rossicus* | accepted | West Kazakhstan and Atyrau regions. | Morphology/regional dataset | No | Historical supported | Include Provisional | [42, 43, 45] | No Kazakhstan tick sequence was identified. |
| *Rhipicephalus leporis* | *Rhipicephalus leporis* | accepted as valid species | West Kazakhstan and Atyrau regions. | Morphology/regional dataset | No | Historical supported / reassessment needed | Need validation | [25, 43] | *Rhipicephalus leporis* is currently recognized as a valid species distinct from *R. linnaei*. Kazakhstan occurrence has been reported from western regions, but the available material has not been re-examined under the current species concept; voucher-based reassessment is therefore required. |
| *Rhipicephalus bursa* | *Rhipicephalus bursa* | accepted | West Kazakhstan and southern regional records. | Morphology | No | Historical supported | Include Provisional | [43] | Primary Kazakhstan material remains to be documented. |
| *Rhipicephalus pumilio* | *Rhipicephalus pumilio* | accepted | West Kazakhstan, Atyrau and the Balkhash region. | Morphology/regional evidence | No | Historical supported | Include Provisional | [37, 42, 43] | Member of the *R. sanguineus* group; modern integrative confirmation is needed. |
| *Rhipicephalus sanguineus s.l.* | *Rhipicephalus sanguineus group* | species-complex flag; historical records disputed | Modern 2016–2024 material lacks lineage resolution; Galuzo rejected earlier local identifications. | Morphology | No | Questionable taxon | Pending Taxonomy | [12, 15, 38] | *Sensu lato* records cannot be assigned to *R. sanguineus* sensu stricto without a lineage-level audit. |
| *Boophilus calcaratus* | *Rhipicephalus microplus complex* | obsolete combination / likely misapplied | Reported from southern Kazakhstan in legacy material. | Morphology using historical keys | No | Questionable taxon | Exclude Until Verified | [26] | Likely misapplied within the *R. microplus* complex; do not merge automatically with *R. annulatus.* |
| *Argas persicus* | *Argas persicus* | accepted | Southern/south-eastern Kazakhstan; 1,651 specimens in a modern dataset. | Morphology + *COI* | Yes | Molecularly confirmed | Include | [18, 38] | COI confirms Kazakhstan material; pathogen detection was not used as tick identification. |
| *Ornithodoros tartakovskyi* | *Ornithodoros tartakovskyi* | accepted | Western, southern and south-eastern Kazakhstan; 36 specimens in 2024. | Morphology; 36 specimens in 2024 | No | Verified morphology | Include | [18, 37, 43] | Modern morphology confirms occurrence, but Kazakhstan tick sequences remain unavailable. |
| *Ornithodoros / Alveonasus lahorensis* | *Alveonasus lahorensis* | genus normalization | Historical Kazakhstan range evidence, principally from southern sources. | Morphology/historical evidence | No | Historical supported | Include Provisional | [15,25] | Generic placement has changed; a modern Kazakhstan voucher is needed. |
| *Ornithodoros papillipes* | *Ornithodoros papillipes* | accepted | Kazakhstan; historical primary occurrence record, with additional host/ecological evidence from natural foci. | Historical morphology / taxonomic identification | No | Historical supported | Include Provisional | [46, 47] | A valid species with direct historical Kazakhstan records; modern voucher-linked and molecular confirmation is lacking. |
| *Ornithodoros coniceps* | *Alectorobius coniceps* | genus reassignment | Kazakhstan; historical primary occurrence record. | Historical morphology / taxonomic identification | No | Historical supported | Include Provisional | [48] | Historical Kazakhstan material was reported as *O. coniceps*; the name is normalized to *Alectorobius coniceps* according to the current classification. Modern confirmation is lacking. |
| *Ornithodoros cholodkovskyi* | *Ornithodoros cholodkovskyi* | accepted | Kazakhstan-specific primary occurrence evidence not recovered. | Literature / taxonomic record | No | Insufficient occurrence evidence | Pending Primary | [49] | Taxonomic validity alone does not demonstrate occurrence in Kazakhstan; no qualifying Kazakhstan-specific primary record was recovered. |
| *Argas vespertilionis* | *Carios vespertilionis* | genus normalization | Southern Kazakhstan; bats and roosts. | Morphology; historical host material | No | Historical supported | Include Provisional | [49] | Now placed in *Carios*; possible cryptic lineages require Kazakhstan molecular material. |
| *Haemaphysalis sulcata* | *Haemaphysalis sulcata* | accepted; species-complex caution | Repeated modern collections in Turkistan, Zhambyl, Almaty and Jetisu regions; Kazakhstan cox1-confirmed material is available from Turkistan Region. | Morphology + *COI/cox1* | Yes | Molecularly confirmed / taxonomic caution | Include Provisional | [18, 23, 33, 39] | Kazakhstan *cox1* evidence is available, but limited sequence representation and possible species-complex structure justify continued provisional treatment. |
| *Haemaphysalis pospelovashtromae* | *Haemaphysalis pospelovashtromae* | accepted | North-western Jetisu Alatau; one collection specimen from spring 2016. | Morphology; institutional collection | No | New/possible record | Include Provisional | [58] | A valid species, but the national record rests on one specimen and needs replication. |
| *Argas reflexus* | *Argas reflexus* | accepted | Almaty; 14 specimens collected from pigeons in 2021–2023. | Morphology using taxonomic keys | No | Verified morphology | Include | [18] | A precise modern specimen-based record independent of the historical Kazakhstan material reassigned to *A. vulgaris* by Filippova; *Borrelia* PCR was not treated as tick molecular identification. |
| *Ixodes stromi* | *Ixodes stromi* | accepted | Zhambyl Region; one female from a wood mouse, 2023–2024. | Morphology | No | New/possible record | Include Provisional | [44] | A single record of a valid species; repeat collection and voucher-linked sequencing are needed. |
| *Haemaphysalis erinacei taurica* | *Haemaphysalis erinacei* | collapse subspecific/legacy name | Zhambyl Region; burrows of small mammals. | Morphology | No | Synonymized/legacy usage | Do Not Count Separately | [44] | Legacy subspecific name retained for traceability but not counted separately. |
| *Argas tridentatus* | *Argas tridentatus* | accepted | Northern Betpak-Dala and Zaisan area; historical Kazakhstan occurrence evidence. | Historical morphology / taxonomic monograph | No | Historical supported | Include Provisional | [49] | A valid species with Kazakhstan-specific historical occurrence evidence; modern voucher-linked and molecular confirmation is lacking. |
| *Argas vulgaris* | *Argas vulgaris* | accepted | Historical Kazakhstan collection material; specimens previously reported as *A. reflexus* were re-examined and assigned to A. vulgaris by Filippova. | Historical morphology / taxonomic re-examination | No | Historical supported | Include Provisional | [49] | *Argas vulgaris* is currently recognized as a valid species. Filippova’s re-examination of Kazakhstan collection material supports historical occurrence, but modern voucher-linked and molecular confirmation is lacking. |
| *Ornithodoros capensis* | *Alectorobius capensis* | genus reassignment; valid species | Historical report from Barsa-Kelmes Island, Aral Sea; primary material requires direct reassessment. | Historical morphology / literature record | No | Historical / validation required | Need validation | [25, 49] | *Alectorobius capensis* is currently recognized as a valid species. Historical literature reports O. capensis from Barsa-Kelmes Island, but the original Kazakhstan material has not been re-examined under the current species concept; the record is therefore retained transparently as requiring validation. |
